# Serum physiology, fecal microbiota, and metabolome signatures associated with perinatal stage transitions in Mongolian mares

**DOI:** 10.3389/fvets.2026.1922191

**Published:** 2026-08-26

**Authors:** Yuanyi Liu, Qianqian He, Gen Wang, Zhenyou Wu, Ming Du, Dongyi Bai, Manglai Dugarjaviin, Xinzhuang Zhang

**Affiliations:** 1 Key Laboratory of Equus Germplasm Innovation, Ministry of Agriculture and Rural Affairs, Hohhot, China; 2 Inner Mongolia Key Laboratory of Equine Science Research and Technology Innovation, Inner Mongolia Agricultural University, Hohhot, China; 3 College of Animal Science, Inner Mongolia Agricultural University, Hohhot, China

**Keywords:** fecal metabolome, fecal microbiota, Mongolian mare, perinatal adaptation, perinatal stage transition

## Abstract

**Introduction:**

Lactation is a critical reproductive trait that influences offspring survival and livestock productivity in equine production. Characterizing physiological transitions during the perinatal period can improve our understanding of how mares adapt to late gestation, parturition, and early lactation. However, in horses—a typical monogastric herbivore with unique digestive characteristics—the associations between gut microbiota and perinatal physiological adaptation, as well as related stage-associated microbial and metabolic features, remain largely uncharacterized.

**Methods:**

In this study, parallel profiling of serum physiology, fecal microbiota, and fecal metabolome was performed to identify candidate stage-associated microbial and metabolic features across three perinatal stages (pre-foaling, PF; post-foaling, PoF; early lactation, EL) in 19 multiparous Mongolian mares selected from a local breeding herd with records of normal foaling and healthy weaning. We further systematically determined serum biochemical indicators, reproductive hormones and immune parameters to interpret host physiological adaptive characteristics linked to lactogenesis.

**Results:**

Obvious stage-specific physiological patterns were observed: triglycerides, urea and mineral elements were present at higher serum concentrations during the pre-foaling period, which may support fetal gestation; serum glucose was higher after parturition, which may meet the energy demands of delivery and early lactation; serum IgA concentration was higher in early lactation, which may be consistent with enhanced mucosal immune activation. Of note, serum IgA does not directly reflect colostral IgA concentrations or foal passive immunity. Alpha diversity of fecal microbiota was lower during the post-foaling stage, and each perinatal stage was associated with distinct fecal microbial community structures. Microbial taxa potentially associated with carbohydrate fermentation were more abundant in the pre-foaling period; the post-foaling stage was associated with an increased relative abundance of *Akkermansia*, which is consistent with a possible role in intestinal barrier maintenance, along with increased PICRUSt2-predicted abundance of galactose metabolism pathways; microbial taxa potentially related to lipid metabolism and bile secretion features were predominant in early lactation.

**Discussion:**

This study systematically characterized stage-specific remodeling patterns of fecal microbiota and fecal metabolome during the perinatal transition in Mongolian mares. The identified fecal microbial taxa and metabolites are described as candidate stage-associated discriminatory features rather than validated biomarkers.

## 1 Introduction

Lactation is the most essential biological function in mammals, and its successful initiation directly determines offspring survival, growth, and development (1). For livestock production, lactation-related traits are closely associated with offspring survival and production efficiency. Characterizing perinatal physiological adaptation can help optimize feeding management and improve reproductive health in equine production. In the equine industry, insufficient milk production is a major constraint that causes high rates of foal mortality and poor growth (2, 3). Therefore, characterizing perinatal physiological and microbial dynamics is important for improving mare health, reducing feeding costs, and optimizing equine production management.

The perinatal period is characterized by dramatic physiological changes, including mammary gland development, hormonal shifts, energy metabolism remodeling, and immune adaptation (4). In recent years, accumulating evidence has indicated that the gut microbiota is widely associated with host metabolism, endocrine status, immune homeostasis, and mammary gland physiology (5, 6). The gut microbiota may influence host physiology through metabolic products such as bile acids, amino acid derivatives, short-chain fatty acids, and other small molecules, forming a microbiota-metabolite axis that may be involved in perinatal physiological adaptation (7, 8). Such coordinated microbial and metabolic signatures are considered promising candidate features for evaluating physiological states during the perinatal period in horses.

In dairy cows, goats, and other ruminants, the gut microbiota has been reported to enhance energy harvest, improve lipid absorption, and promote milk fat synthesis (9). Multiple fecal microbial and metabolic features associated with lactation stages have been identified in ruminants, providing references for precision feeding management. However, horses are monogastric herbivores with unique digestive physiology, and the dynamic changes of fecal microbiota during lactation, as well as stage-associated microbial and metabolic features in horses, remain poorly understood (10). Mongolian horses are a hardy local breed that exhibits strong adaptability to harsh environments and stable reproductive performance under conditions including low temperature, limited nutrient availability, and high grazing stress (11). However, the physiological and microbial dynamics during the perinatal period have not been systematically investigated in this breed, and stage-associated microbial and metabolic signatures during perinatal adaptation remain poorly characterized.

We hypothesized that during the perinatal period, Mongolian mares undergo dynamic remodeling of fecal microbiota and metabolome, which are coordinately associated with host energy metabolism, hormone secretion, and immune function during perinatal adaptation (12). Therefore, this study performed parallel multi-omic profiling of serum physiology, fecal microbiota, and fecal metabolome across three perinatal stages. The objectives were: (1) to characterize dynamic changes in host physiological phenotypes during lactation initiation; (2) to identify candidate stage-associated fecal microbial and metabolic features related to perinatal adaptation; (3) to characterize stage-specific shifts in gut microbiota, fecal metabolites and host physiological phenotypes associated with lactation adaptation. This study provides a comprehensive overview of physiological and fecal microbial adaptive patterns during the perinatal transition in Mongolian mares, and supplies valuable reference information for perinatal nutritional regulation and germplasm conservation in equine production, offering new insights into perinatal physiological adaptation in monogastric herbivores.

## 2 Materials and methods

### 2.1 Animals and experimental design

All experimental procedures were conducted in strict accordance with the guidelines for the care and use of experimental animals approved by the Laboratory Animal Welfare and Ethics Committee of Inner Mongolia Agricultural University (No. NND2024009). The study was carried out at the White Horse Base in Xiwuzhumuqin Banner, Xilingol League, Inner Mongolia Autonomous Region, China. Nineteen healthy multiparous Mongolian mares (12–16 years of age; 250 ± 25 kg body weight; parity 5–8) were enrolled as experimental subjects. All mares were selected from a local core breeding herd with documented records of normal foaling and healthy weaning of foals; no formal quantitative ranking of lactation performance was performed. All mares were housed under uniform conditions with *ad libitum* access to water and hay; the nutrient composition of the hay is detailed in Supplemental Table S1. Each mare was longitudinally sampled across three perinatal stages: pre-foaling (PF, 1–2 months before foaling), post-foaling (PoF, 1–3 days after foaling), and early lactation (EL, 1 month after foaling).

### 2.2 Sample collection

Blood samples were collected from the jugular vein into sterile coagulation tubes. After standing at room temperature for 30 min, samples were centrifuged at 3,000 × g for 10 min at 4 °C to obtain serum, which was stored at −80 °C. Fresh fecal samples were collected directly from the rectum using sterile gloves, immediately frozen in liquid nitrogen, and stored at −80 °C for microbiota and metabolome analyses (13, 14).

### 2.3 Biochemical parameters

Serum biochemical profiles were comprehensively analyzed using a fully automated veterinary clinical chemistry analyzer (Mindray Bio-Medical Electronics Co., Ltd., Shenzhen, China, Model: BS-180VET). The measured parameters covered three functionally distinct categories: (1) liver and muscle function markers: lactate dehydrogenase (LDH), alanine aminotransferase (ALT), aspartate aminotransferase (AST), creatine kinase (CK), γ-glutamyl transferase (GGT), and alkaline phosphatase (ALP); (2) electrolyte and mineral indicators: calcium (Ca), phosphorus (P), and magnesium (Mg); (3) metabolic and renal function indices: total protein (TP), albumin (ALB), globulin (GLB), albumin-to-globulin ratio (A/G), triglycerides (TG), urea (UREA), glucose (GLU), total cholesterol (TC), and creatinine (CREA) (14–16).

### 2.4 Reproductive hormones

Circulating gonadotropin-releasing hormone (GnRH) was measured using a commercially available equine ELISA kit according to the manufacturer's protocol, to evaluate potential changes in hypothalamic axis activity. GnRH results were interpreted cautiously, as peripheral serum concentrations may not fully reflect hypothalamic secretion dynamics due to pulsatile release and short circulating half-life. A complete panel of reproductive hormones was quantified using species-specific enzyme-linked immunosorbent assay (ELISA) kits (Solarbio Science & Technology Co., Ltd., Beijing, China) that have been thoroughly optimized for equine serum samples. The assayed hormones included hypothalamic-pituitary axis regulators: GnRH, follicle-stimulating hormone (FSH), luteinizing hormone (LH), prolactin (PRL), and oxytocin (OXT); as well as gonadal steroid hormones: estradiol (E2), progesterone (P4), and testosterone (T). All assays were conducted in strict compliance with the manufacturer's standardized operating procedures (14, 17).

### 2.5 Immune indices

Serum immune parameters were determined using commercial ELISA kits manufactured by Inner Mongolia Baoxin Biotechnology Co., Ltd. The assays simultaneously assessed two key components of the immune system: humoral immune markers (immunoglobulin M, IgM; immunoglobulin A, IgA; immunoglobulin G, IgG) and pro-inflammatory cytokines (interleukin-6, IL-6; tumor necrosis factor-α, TNF-α; interleukin-1β, IL-1β; interleukin-2, IL-2). All experimental steps were performed exactly as specified in the kit protocols to ensure assay reliability, reproducibility, and consistency across all samples (14, 18).

### 2.6 Fecal microbiome sequencing protocol

Total genomic DNA was isolated from fecal specimens using a commercial spin-column kit (Solarbio Science & Technology Co., Ltd., Beijing, China, Cat. No. D2700) following the manufacturer's standard protocol. The V3-V4 hypervariable region of the bacterial 16S rRNA gene was amplified using barcoded fusion primers to enable multiplexed sequencing. PCR amplicons were first size-selected via 2% agarose gel electrophoresis, then further purified using magnetic beads to eliminate primer dimers and non-specific amplification products. Purified amplicons were ligated to Illumina dual-index adapters with T4 DNA ligase, and library quality was verified using an Agilent 2100 Bioanalyzer prior to pooling and paired-end sequencing on the Illumina NovaSeq 6000 platform.

### 2.7 Fecal microbiome data processing pipeline

Raw sequencing reads in FastQ format were subjected to rigorous multi-step quality control using FASTP (v0.23.2). Reads were trimmed to remove adapter sequences and low-quality bases (Phred quality score < 20 across >50% of the read length). Paired-end reads were then assembled into contigs using FLASH (v1.2.11) with a minimum overlap of 10 base pairs and a maximum mismatch rate of 0.1. Chimeric sequences were detected and removed using the UCHIME algorithm implemented in USEARCH (v11.0.667) against the Gold reference database.

High-quality clean reads were clustered into OTUs at the 97% similarity threshold using the UPARSE pipeline, and singleton OTUs were discarded to reduce artificial inflation of diversity estimates. For alpha diversity calculations, all samples were rarefied to an equal sequencing depth to account for differences in read counts. For differential abundance analysis of microbial taxa, OTU abundance tables were normalized using the median ratio method implemented in DESeq2 to correct for uneven sequencing depth across samples.

### 2.8 Fecal microbiome bioinformatic analysis

Microbial community structure was characterized using a comprehensive analytical framework comprising six complementary approaches: (1) Alpha diversity indices, including Shannon index, observed OTUs, Sob index, Chao1 index, ACE index, and PD-tree index, were calculated using QIIME2 (v2022.2) after rarefaction to 20,000 reads per sample; (2) Beta diversity was primarily visualized using unsupervised principal coordinate analysis (PCoA) based on Bray-Curtis dissimilarity. Statistical differences in community structure between stages were assessed using PERMANOVA (Adonis test). PERMDISP analysis was performed to evaluate differences in multivariate dispersion between groups. Supervised partial least squares discriminant analysis (PLS-DA) was performed as an additional exploratory approach; (3) Taxonomic classification of representative OTU sequences was performed using the RDP classifier against the SILVA 138.1 database with a confidence threshold of 0.8; (4) Differential abundance analysis was conducted using LEfSe (LDA score threshold >2, an exploratory feature discovery tool) to identify stage-associated microbial taxa.; (5) Functional potential of the gut microbiome was predicted using PICRUSt2 based on KEGG Orthology pathways; (6) Statistical significance of functional pathway differences was determined using STAMP software with Welch's *t*-test (*p* < 0.05).

### 2.9 Fecal metabolomics sample preparation

Metabolite extraction was performed using a modified methyl tert-butyl ether (MTBE) dual-phase extraction protocol optimized for fecal matrices (Majorbio Bio-pharmaceutical Technology Co., Ltd., Shanghai, China), which enables simultaneous recovery of both polar and non-polar metabolites. Briefly, 100 mg of lyophilized fecal powder was fortified with 10 μL of an internal standard mixture (10 μM each of 13C-labeled amino acids and deuterated lipids). Samples were then homogenized by vortexing for 3 min with 1 mL of ice-cold solvent mixture (methanol:MTBE:water, 1:3:1, v/v/v). This solvent system was selected for its superior ability to partition polar metabolites into the lower methanol-water phase and non-polar metabolites into the upper MTBE phase.

After centrifugation at 12,000 × g for 10 min at 4 °C, both the upper organic phase and lower aqueous phase were carefully transferred to the same centrifuge tube and evaporated to dryness under a gentle stream of nitrogen gas. The dried residue was reconstituted in 200 μL of 50% acetonitrile containing 0.1% formic acid, filtered through a 0.22 μm membrane filter, and transferred to LC-MS vials for analysis. This optimized protocol achieved >85% recovery for representative test compounds while minimizing matrix effects.

### 2.10 Untargeted metabolomics analytical conditions

Chromatographic separation was performed on a Waters ACQUITY UPLC HSS T3 column (2.1 × 100 mm, 1.8 μm) maintained at 40 °C. The mobile phase consisted of 0.1% formic acid in water (phase A) and 0.1% formic acid in acetonitrile (phase B). The gradient elution program was as follows: 5% B (0–1 min), linear increase to 95% B (1–9 min), hold at 95% B (9–11 min), then return to 5% B (11–12 min) for column re-equilibration. The flow rate was set at 0.3 mL/min, and the injection volume was 2 μL.

Mass spectrometric detection was carried out on a Q Exactive HF-X hybrid quadrupole-Orbitrap mass spectrometer (Thermo Fisher Scientific) operating in both positive (ESI+) and negative (ESI–) electrospray ionization modes. The key MS parameters were: spray voltage 3.5 kV (ESI+) and 2.5 kV (ESI–), capillary temperature 320 °C, sheath gas flow rate 40 arb, auxiliary gas flow rate 10 arb. Full scan spectra were acquired over the m/z range 70-1050 at a resolution of 120,000. Data-dependent acquisition (DDA) was used to select the top 10 most abundant precursor ions per scan cycle for MS/MS fragmentation with normalized collision energies of 20, 30, and 40 eV.

### 2.11 Metabolomics data preprocessing and quality control

Both positive ion mode (POS) and negative ion mode (NEG) were employed for untargeted metabolite detection to maximize metabolite coverage, and data from the two ionization modes were processed and analyzed separately in all subsequent bioinformatics steps.

Quality control (QC) samples were prepared by pooling equal volumes from all experimental samples, and were injected every 10 experimental samples throughout the entire analytical run to continuously monitor instrumental stability and signal reproducibility (19). Principal component analysis (PCA) was conducted using the gmodels package (v2.18.1) in R to evaluate the overall data quality; tight clustering of all QC samples in the PCA score plot confirmed high analytical stability and reliable data quality (https://CRAN.R-project.org/package=gmodels).

Raw mass spectrometry data were subjected to peak picking, retention time alignment and feature grouping using the XCMS package with standard optimized parameters. Standard peak filtering was performed to remove low-quality and unstable features prior to statistical analysis, and procedural blank samples were processed in parallel for background subtraction, following the standard untargeted metabolomics workflow.

Missing values in the data matrix were imputed using the k-nearest neighbors (k-NN) algorithm with k = 5, which is a widely adopted standard method for untargeted metabolomics data that effectively preserves the biological variation of metabolite abundance. All samples were analyzed in a single analytical batch with a randomized injection order, with QC samples inserted at regular intervals to mitigate systematic signal drift; thus no additional batch correction was performed in this study.

Metabolite annotation was carried out by matching accurate mass and retention time against an in-house authentic reference standard library, and the identities of key candidate metabolites were further validated by manual inspection of MS/MS fragmentation spectra, following standard identification criteria for untargeted metabolomics.

For functional analysis, differential metabolites were mapped to the KEGG database for pathway annotation and enrichment analysis.

### 2.12 Statistical analysis

Differential metabolites were screened by combining multivariate and univariate statistical criteria. Orthogonal partial least squares discriminant analysis (OPLS-DA) was conducted using the ropls package in R with 7-fold cross-validation, and variable importance in projection (VIP) scores were extracted. Univariate statistical analysis was performed using paired Student's *t*-test to match the longitudinal study design. Metabolites with VIP ≥ 1 and *p* < 0.05 were defined as significantly differential metabolites. A model with *Q*^2^ > 0.5 was considered to have good predictive ability (20).

Pathway enrichment analysis was performed using MetaboAnalyst 5.0, where metabolites were mapped to KEGG metabolic pathways, and over-representation *p*-values were calculated using the hypergeometric test. All statistical analyses were performed in R (v4.1.2), and data visualization was generated using the ggplot2 package. Reproducibility was ensured by setting a random seed [set.seed(123)] for all randomization-based analyses (21).

Initial data organization was performed using Microsoft Excel (Microsoft Corporation, Redmond, WA, USA). Data are presented as mean ± standard deviation (Mean ± SD) to describe the distribution of individual observations. Additional statistical analyses were conducted using SPSS software (version 27.0). Repeated-measures analysis of variance (ANOVA) was used to compare physiological parameters and alpha diversity indices across the three perinatal stages, to properly account for the longitudinal repeated-measures design. Mauchly's test was used to verify the sphericity assumption. Tukey's Honestly Significant Difference (HSD) *post-hoc tes*t was used for subsequent pairwise comparisons between stages. For microbial differential abundance analysis at each taxonomic level, Welch's *t*-test was used for exploratory pairwise comparisons between stages (14, 22).

## 3 Results

### 3.1 Serum biochemical parameters during the perinatal period

**Figure 1:**
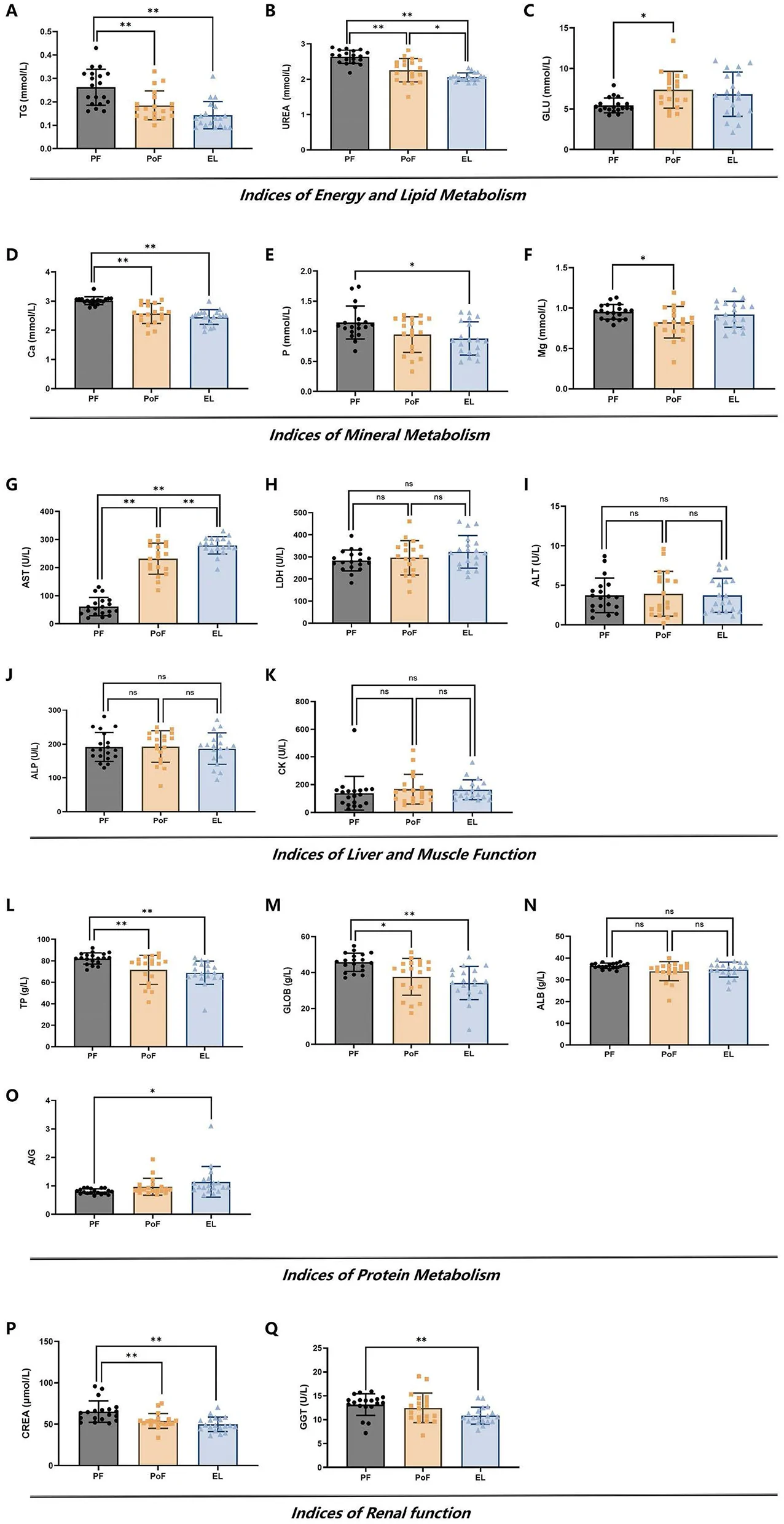
Serum biochemical parameters of Mongolian mares across three perinatal stages. Pairwise comparisons were performed using Tukey's HSD *post-hoc* test following repeated-measures ANOVA. **(A)** Triglyceride (TG) concentration; **(B)** Urea (UREA) concentration; **(C)** Glucose (GLU) concentration; **(D)** Calcium (Ca) concentration; **(E)** Phosphorus concentration; **(F)** Magnesium (Mg) concentration; **(G)** Aspartate aminotransferase (AST) activity; **(H)** Lactate dehydrogenase (LDH) activity; **(I)** Alanine aminotransferase (ALT) activity; **(J)** Alkaline phosphatase (ALP) activity; **(K)** Creatine kinase (CK) activity; **(L)** Total protein (TP) concentration; **(M)** Globulin (GLOB) concentration; **(N)** Albumin (ALB) concentration; **(O)** Albumin-to-globulin (A/G) ratio; **(P)** Creatinine (CREA) concentration; **(Q)** γ-glutamyl transferase (GGT) activity. *p < 0.05, **p < 0.01; ns, not significant. *n* = 19.

Serum biochemical indices exhibited highly stage-specific changes, which may be associated with metabolic adaptations during the perinatal transition. Changes in all serum biochemical indices are presented in Figure 1. Detailed raw data are provided in Supplemental Table S2.

Energy and lipid metabolism-related parameters are shown in Figures 1A–C: Triglyceride (TG) concentrations were significantly higher in the PF period than in the PoF and EL periods (*p* < 0.01), and decreased continuously after parturition (Figure 1A). Blood urea (UREA) showed a persistent downward trend from PF to EL (*p* < 0.01). This may reflect altered protein metabolism around parturition, potentially including changes in dietary intake, hepatic metabolism, hydration status, or renal handling (Figure 1B). Glucose (GLU) peaked at PoF and remained at a relatively high level during EL, with significant intergroup differences observed (*p* < 0.05). This pattern may support the energy requirements of parturition and early lactation (Figure 1C).

Mineral metabolism parameters are presented in Figures 1D–F: Calcium (Ca), phosphorus (P), and magnesium (Mg) all decreased significantly after parturition. Serum calcium levels were significantly higher in PF than in PoF and EL (*p* < 0.01); phosphorus and magnesium concentrations were significantly lower in PoF and EL compared with PF (*p* < 0.05), which may be associated with mineral mobilization for fetal development and perinatal physiological needs.

Liver and muscle function parameters are shown in Figures 1G–K: Aspartate aminotransferase (AST) increased continuously and significantly from PF to EL (*p* < 0.01) (Figure 1G), indicating mild muscle damage and physiological stress induced by parturition. Lactate dehydrogenase (LDH), alanine aminotransferase (ALT), alkaline phosphatase (ALP), and creatine kinase (CK) showed no significant differences across the three stages (*p* > 0.05), suggesting stable hepatic function during the perinatal period (Figures 1H–K).

Protein metabolism parameters are presented in Figures 1L–O: Total protein (TP) and globulin (GLOB) decreased significantly after parturition (*p* < 0.01) (Figures 1L, M), while albumin (ALB) levels showed a trend toward statistical significance (*p* = 0.064) (Figure 1N). The albumin-to-globulin ratio (A/G) increased significantly from PF to EL (*p* < 0.05) (Figure 1O), indicating adaptive redistribution of protein synthesis for lactation and reduced systemic protein catabolism.

Renal function parameters are shown in Figures 1P, Q: Creatinine (CREA) decreased continuously (*p* < 0.01), and γ-glutamyl transferase (GGT) was significantly lower in EL than in PF (*p* < 0.01), reflecting adaptive adjustments in renal metabolism during the perinatal period.

### 3.2 Reproductive hormone indices during the perinatal period

**Figure 2:**
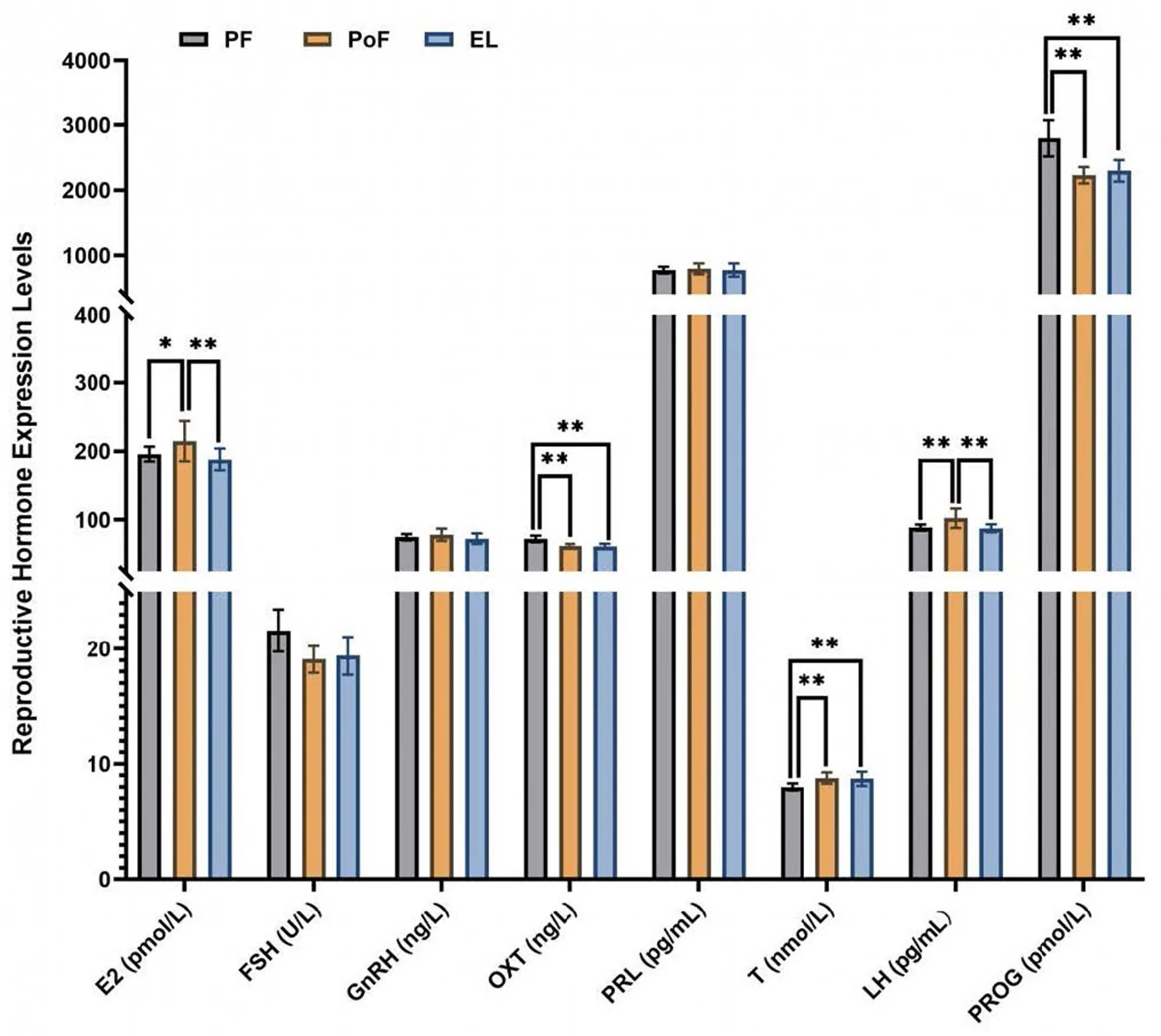
Serum reproductive hormone levels of Mongolian mares across three perinatal stages. Pairwise comparisons were performed using Tukey's HSD *post-hoc* test following repeated-measures ANOVA. *p < 0.05, **p < 0.01. *n* = 19.

The indices related to reproductive hormones are illustrated in Figure 2. Progesterone concentration was highest in PF and dropped sharply after foaling (*p* < 0.01), relieving the inhibition of lactation. Estradiol peaked in PoF (*p* < 0.01), promoting mammary gland development and lactation initiation. Testosterone was significantly higher in PoF and EL (*p* < 0.01), suggesting a less well-characterized role in energy metabolism and lactation maintenance. Oxytocin was higher in PF to promote parturition and then decreased. LH peaked in PoF, indicating transient activation of the hypothalamic-pituitary-gonadal axis. Detailed raw data are provided in Supplemental Table S3.

### 3.3 Immune indices during the perinatal period

**Figure 3:**
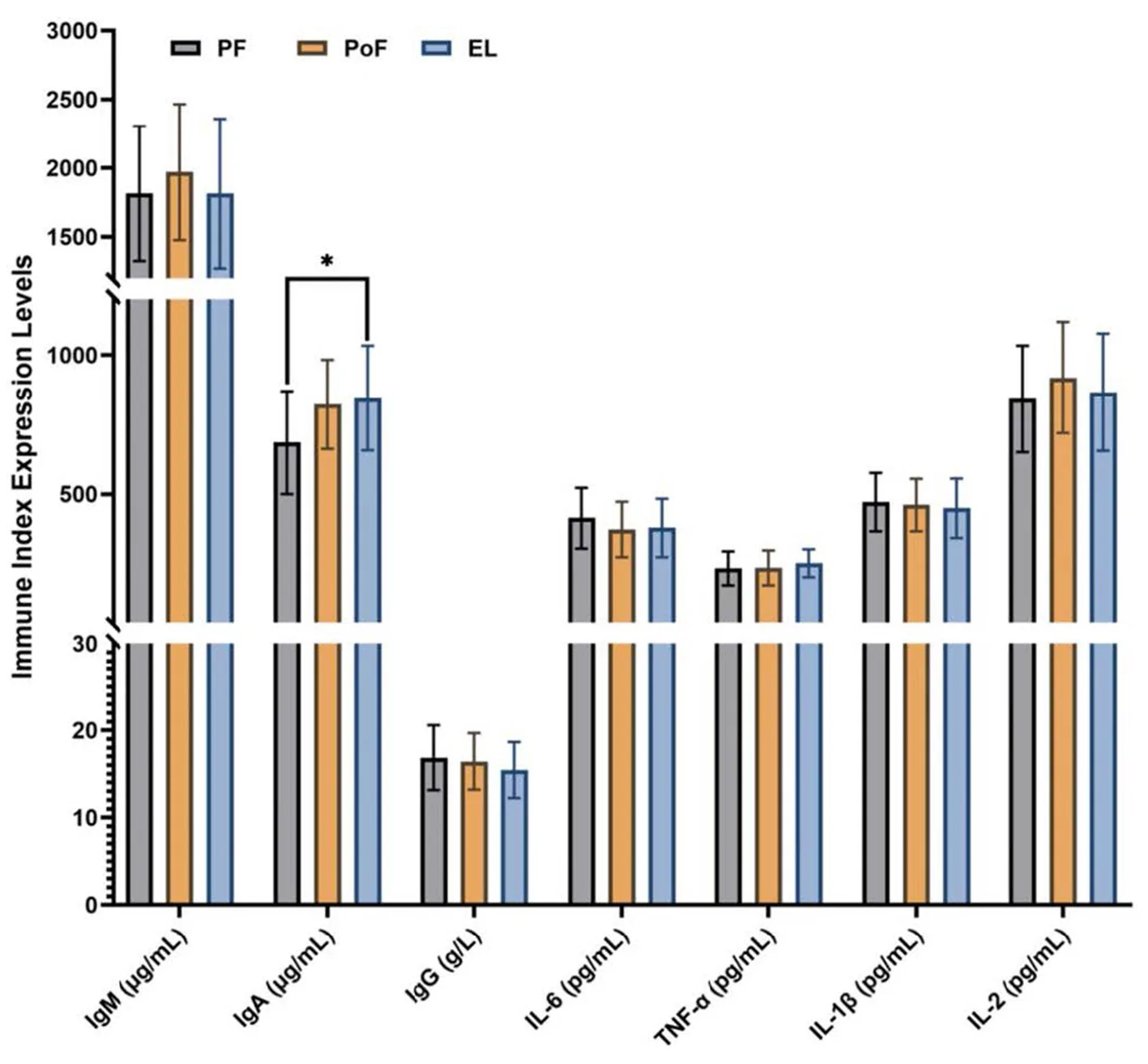
Serum immune index levels of Mongolian mares across three perinatal stages. Pairwise comparisons were performed using Tukey's HSD *post-hoc* test following repeated-measures ANOVA. *p < 0.05. *n* = 19.

Most immune indices remained stable across the perinatal period. Notably, IgA concentration was higher in EL (*p* < 0.05), which may be associated with enhanced mucosal immune activation. Of note, serum IgA does not directly reflect colostral IgA concentrations. IgG, IgM, and cytokines including IL-6, TNF-α, IL-1β, and IL-2 showed no significant changes, indicating stable immune homeostasis without excessive inflammation or immunosuppression (Figure 3). Detailed raw data are provided in Supplemental Table S4. In summary, the immune system of perinatal Mongolian mares remains in a stable homeostatic state overall, with only mucosal immune-related IgA showing a stage-specific increase in early lactation.

### 3.4 Alpha diversity of fecal microbiota during the perinatal period

**Figure 4:**
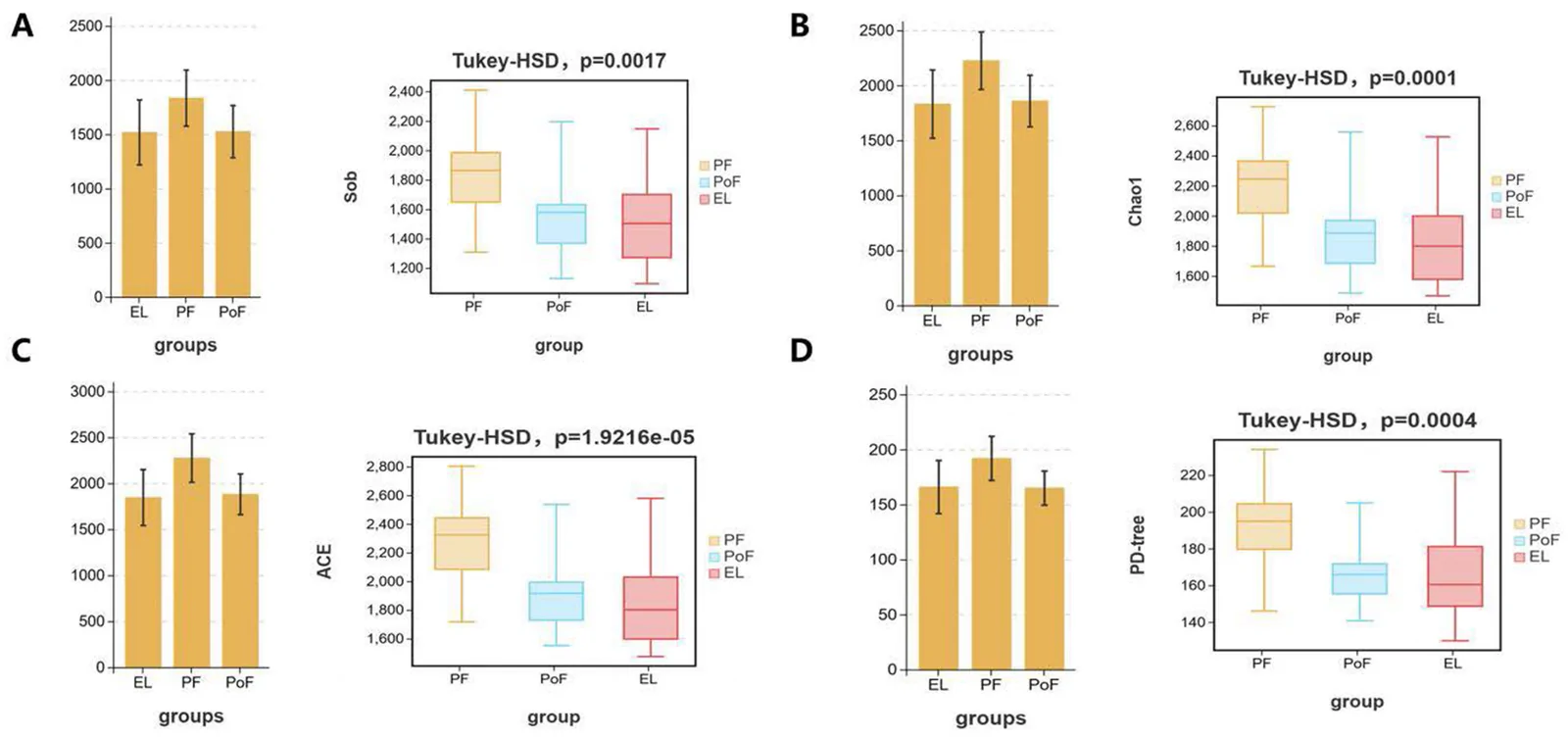
Alpha diversity analysis of gut microbiota in Mongolian mares across three perinatal stages (*n* = 19). **(A)** Sobs index; **(B)** Chao1 index; **(C)** ACE index; **(D)** PD-tree index. Each panel includes a bar chart showing the mean value and a box plot showing the distribution of the index, and all indices showed statistically significant differences among stages (*p* < 0.01).

Figure 4 illustrates the alpha diversity characteristics of the gut microbiota across three perinatal stages (PF, PoF, and EL) in Mongolian mares, including four core indices: species richness index (Sob, Figure 4A), Chao1 index (Figure 4B), ACE index (Figure 4C), and phylogenetic diversity index (PD-tree, Figure 4D). All intergroup differences were statistically analyzed using the Tukey-HSD test.

As shown in Figure 4A, the Sob index was highest in the PF stage, decreased significantly in the PoF stage, and rebounded slightly in the EL stage but remained lower than that in the PF stage, with statistically significant differences among the three stages (*p* = 0.0017). The Chao1 index (Figure 4B) exhibited an identical trend to the Sob index, with the PF stage showing significantly higher values than the PoF and EL stages (*p* = 0.0001), and no significant difference was observed between the PoF and EL stages. The ACE index (Figure 4C) also followed the pattern of being highest in PF, lowest in PoF, and recovered in EL, with extremely significant differences among the three stages (*p* < 0.001). The phylogenetic diversity PD-tree index (Figure 4D) adhered to the same change pattern, with the PF stage significantly higher than the PoF and EL stages (*p* < 0.001), and the PD-tree index in the EL stage was slightly higher than that in the PoF stage. In summary, the alpha diversity of the gut microbiota in Mongolian mares decreased significantly after parturition (PoF) and began to recover gradually during early lactation (EL), but had not yet returned to the level observed in the pre-foaling period (PF). These results indicate that alpha diversity of fecal microbiota is lower during the post-foaling stage, and gradually recovers during early lactation. The underlying mechanisms may include perinatal hormonal fluctuations and physiological stress.

### 3.5 Beta diversity of fecal microbiota during the perinatal period

**Figure 5:**
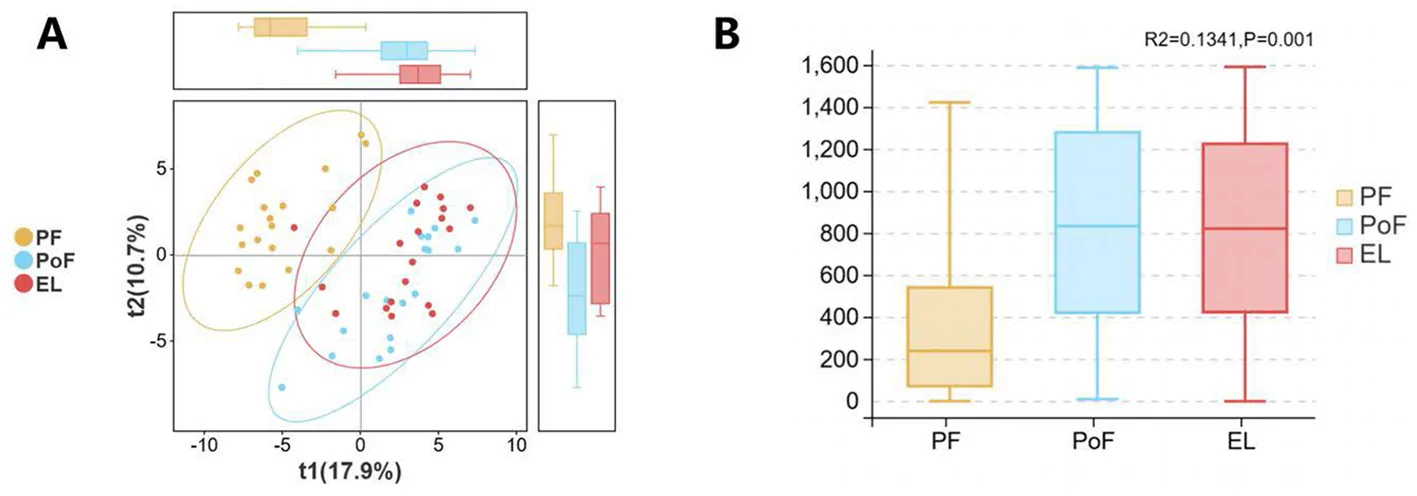
Beta diversity analysis of gut microbiota in Mongolian mares across three perinatal stages (*n* = 19). **(A)** PLS-DA score plot showing the separation of gut microbial community structures among stages; **(B)** Box plot of Bray-Curtis dissimilarity-based community dispersion with Adonis test results.

Figure 5 presents the beta diversity analysis results of gut microbial communities across three perinatal stages (PF, PoF, and EL) in Mongolian mares, including partial least squares discriminant analysis (PLS-DA, Figure 5A) and Adonis test based on Bray-Curtis dissimilarity (Figure 5B). As shown in Figure 5A, the PLS-DA score plot revealed clear clustering and separation of samples among the three stages. The first principal component (t_1_) explained 17.9% of the total variance in community structure, and the second principal component (t_2_) explained 10.7% of the variance, indicating fundamental differences in gut microbial community structure among the three stages. The Adonis test based on Bray-Curtis dissimilarity (Figure 5B) further confirmed the statistical significance of intergroup differences (*p* = 0.001). Meanwhile, the box plot showed that samples in the PF stage had higher community similarity (lower dispersion), while community dispersion was higher in the PoF and EL stages. This pattern indicates greater inter-individual variation in microbial community structure after parturition, and should be interpreted cautiously as it does not necessarily reflect directed biological restructuring.

### 3.6 Compositional characteristics and intergroup differences of gut microbiota at the phylum level during the perinatal period

**Figure 6:**
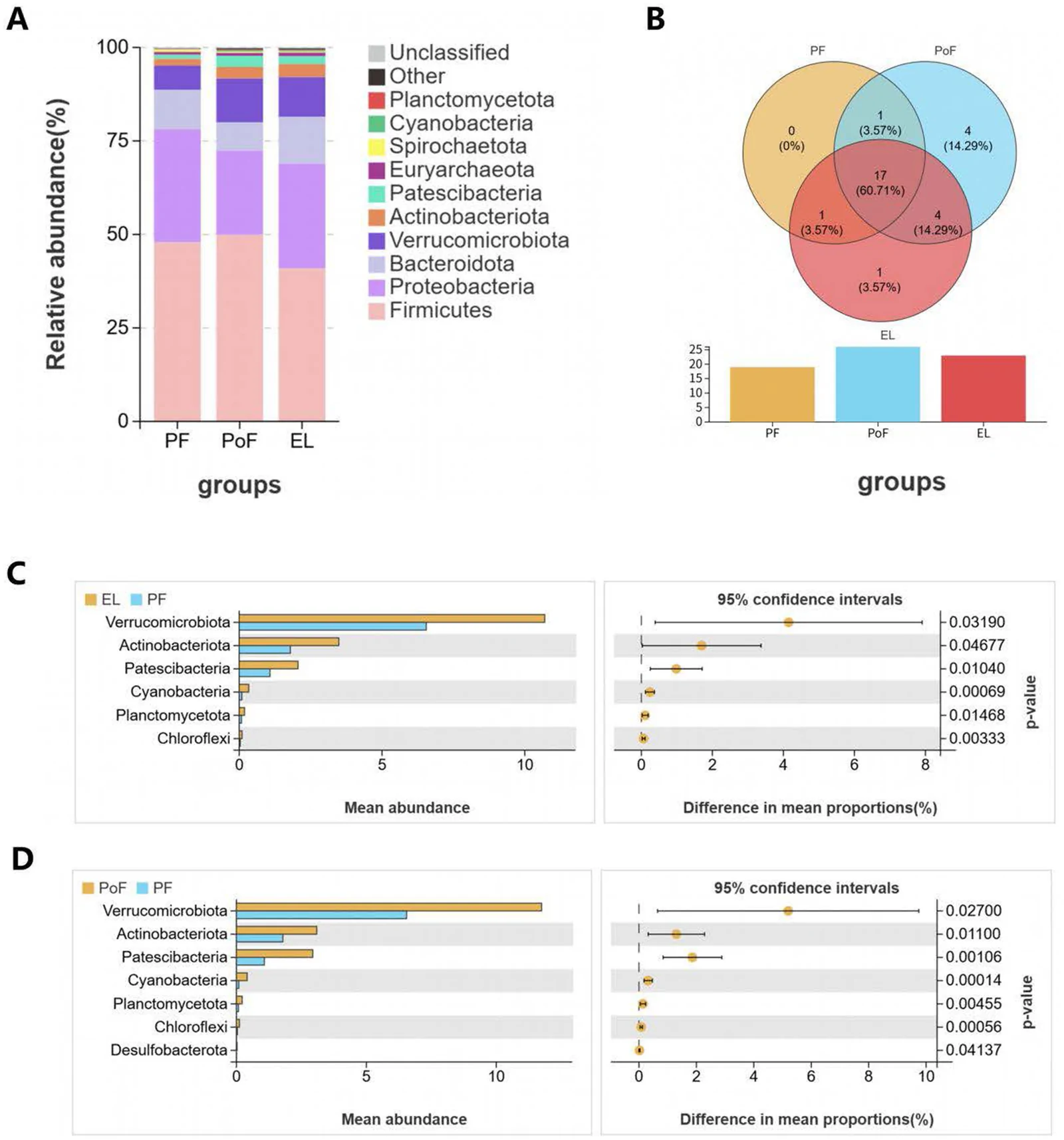
Compositional characteristics and intergroup differences of gut microbiota at the phylum level in perinatal Mongolian mares (*n* = 19). **(A)** Stacked bar chart showing the relative abundance of dominant bacterial phyla in each stage; **(B)** Venn diagram showing shared and unique phyla among the three stages, with the lower bar plot indicating the total number of phyla detected in each stage; the shared core phyla represent stably colonized taxa throughout the perinatal period, while unique phyla reflect stage-specific transient colonization. The lower bar plot indicates the total number of phyla detected in each stage; **(C)** Differential abundance analysis of bacterial phyla between the EL and PF stages using Welch's *t*-test; **(D)** Differential abundance analysis of bacterial phyla between the PoF and PF stages using Welch's *t*-test.

Figure 6 systematically presents the compositional characteristics and differential changes of gut microbiota at the phylum level across three perinatal stages (PF, PoF, and EL) in Mongolian mares.

As shown in Figure 6A, the gut microbiota in all three stages was dominated by *Firmicutes, Proteobacteria*, and *Bacteroidota*, which collectively accounted for more than 85% of the total sequences. Venn diagram analysis in Figure 6B revealed that 17 core phyla were shared among the three stages (accounting for 60.71% of the total phyla); no unique phyla were identified in the PF stage, 4 unique phyla were present in the PoF stage, and 1 unique phylum was found in the EL stage. The bar plot below further indicated that the total number of phyla in both the PoF and EL stages was higher than that in the PF stage after parturition.

The results of intergroup differential analysis are shown in Figures 6C, D. Figure 6C demonstrated that compared with the PF stage, the relative abundances of 6 phyla were significantly increased in the EL stage, namely *Verrucomicrobiota, Actinobacteriota, Patescibacteria, Cyanobacteria, Planctomycetota*, and *Chloroflexi* (*p* < 0.05). Figure 6D showed that in addition to the above 6 phyla, the relative abundance of *Desulfobacterota* was also significantly increased in the PoF stage compared with the PF stage (*p* < 0.05).

### 3.7 Compositional characteristics and intergroup differences of gut microbiota at the class level during the perinatal period

**Figure 7:**
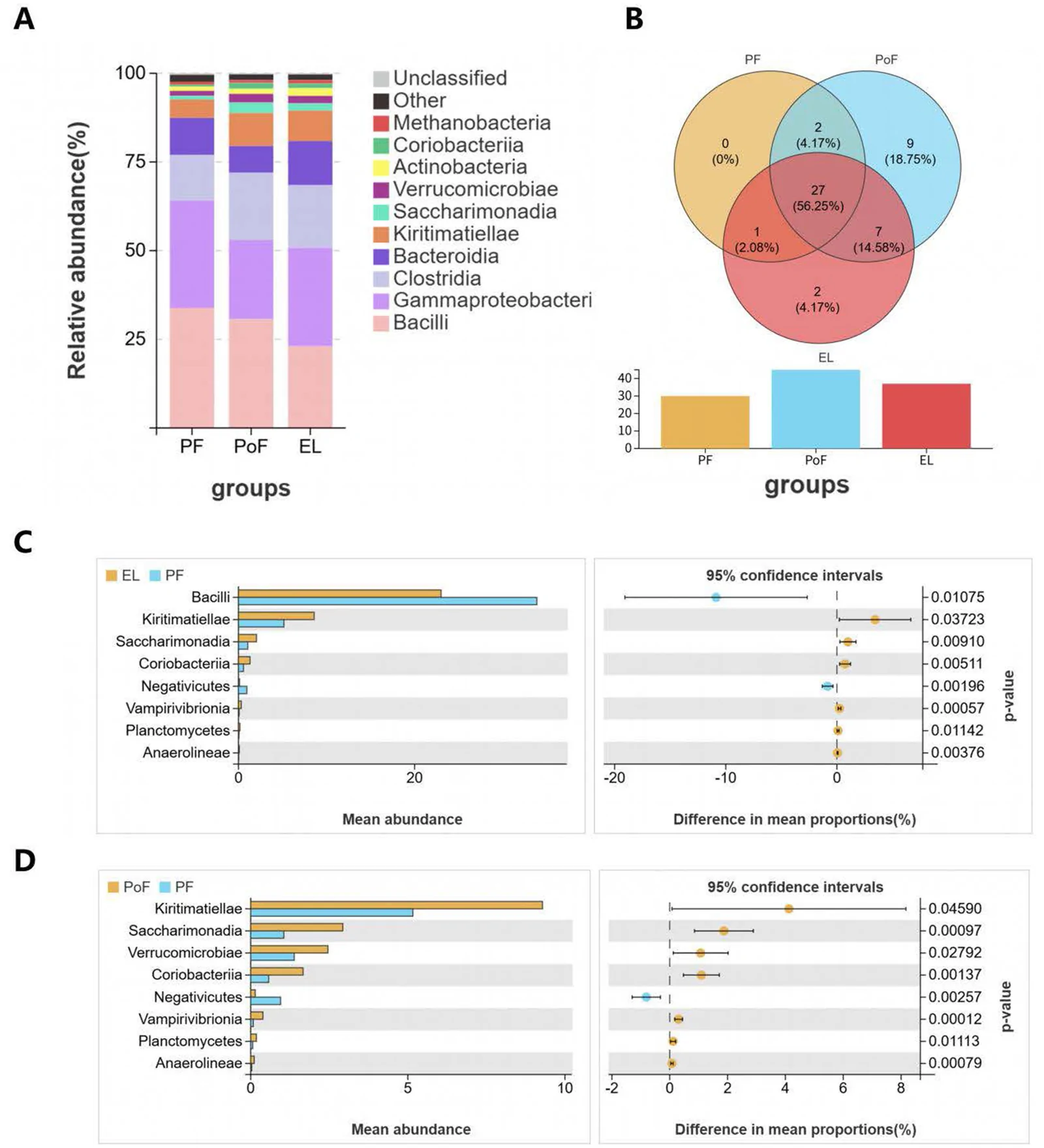
Compositional characteristics and intergroup differences of gut microbiota at the class level in perinatal Mongolian mares (*n* = 19). **(A)** Stacked bar chart showing the relative abundance of dominant bacterial classes in each stage; **(B)** Venn diagram showing shared and unique bacterial classes among the three stages, with the lower bar plot indicating the total number of classes detected in each stage; **(C)** Differential abundance analysis of bacterial classes between the EL and PF stages using Welch's *t*-test; **(D)** Differential abundance analysis of bacterial classes between the PoF and PF stages using Welch's *t*-test.

Figure 7 systematically presents the compositional characteristics and differential changes of gut microbiota at the class level across three perinatal stages (PF, PoF, and EL) in Mongolian mares.

As shown in Figure 7A, the gut microbiota in all three stages was dominated by *Clostridia, Gammaproteobacteria, Bacteroidia*, and *Bacilli*, which collectively accounted for more than 80% of the total sequences. Venn diagram analysis in Figure 7B revealed that 27 core classes were shared among the three stages (accounting for 56.25% of the total classes); no unique classes were identified in the PF stage, nine unique classes were present in the PoF stage, and two unique classes were found in the EL stage. The bar plot below further indicated that the total number of classes was highest in the PoF stage after parturition, followed by the EL stage, and lowest in the PF stage.

The results of intergroup differential analysis are shown in Figures 7C, D. As shown in Figure 7C (EL vs. PF), *Bacilli* was the most abundant core bacterial class in both stages, and its abundance was significantly higher in the PF stage than in the EL stage (*p* < 0.05). *Negativicutes* was also significantly enriched in the PF stage (*p* < 0.05). In contrast, *Kiritimatiellae, Saccharimonadia, Coriobacteriia, Vampirivibrionia, Planctomycetes*, and *Anaerolineae* were all significantly enriched in the EL stage (*p* < 0.05), and the overall abundance of these classes was much lower than that of *Bacilli*.

As shown in Figure 7D (PoF vs. PF), *Kiritimatiellae* was the most abundant bacterial class in both stages, and its abundance was significantly higher in the PoF stage than in the PF stage (*p* < 0.05). The abundance of *Negativicutes* was significantly higher in the PF stage than in the PoF stage (*p* < 0.05). In addition, *Saccharimonadia, Verrucomicrobiae, Coriobacteriia, Vampirivibrionia, Planctomycetes*, and *Anaerolineae* were all significantly enriched in the PoF stage (*p* < 0.05).

### 3.8 Compositional characteristics and intergroup differences of gut microbiota at the genus level during the perinatal period

**Figure 8:**
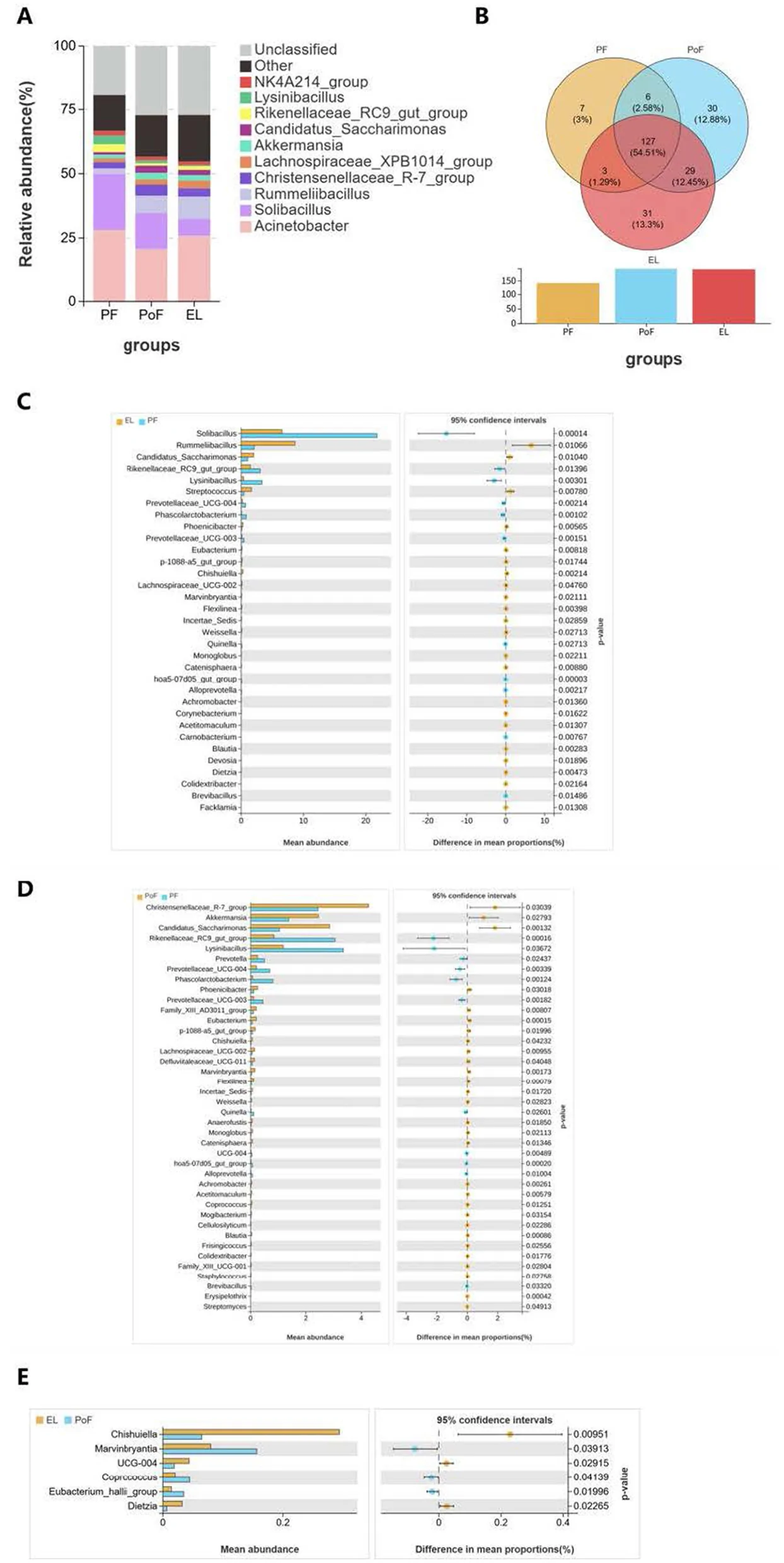
Compositional characteristics and pairwise intergroup differences of gut microbiota at the genus level in perinatal Mongolian mares (*n* = 19). **(A)** Stacked bar chart showing the relative abundance of dominant bacterial genera in each stage; **(B)** Venn diagram showing shared and unique bacterial genera among the three stages, with the lower bar plot indicating the total number of genera detected in each stage; **(C)** Differential abundance analysis of bacterial genera between the EL and PF stages using Welch's *t*-test; **(D)** Differential abundance analysis of bacterial genera between the PoF and PF stages using Welch's *t*-test; **(E)** Differential abundance analysis of bacterial genera between the EL and PoF stages using Welch's *t*-test.

Figure 8 systematically presents the compositional characteristics and pairwise intergroup differential changes of gut microbiota at the genus level across three perinatal stages (PF, PoF, and EL) in Mongolian mares.

As shown in Figure 8A, the gut microbiota in all three stages contained a high proportion of unclassified taxa at the genus level, and the classified dominant genera included *Acinetobacter, Solibacillus*, and *Rummeliibacillus*. Venn diagram analysis in Figure 8B revealed that 127 core genera were shared among the three stages (accounting for 54.51% of the total genera); seven unique genera were identified in the PF stage, 30 unique genera were present in the PoF stage, and 31 unique genera were found in the EL stage. The bar plot below further indicated that the total number of genera in both the PoF and EL stages was significantly higher than that in the PF stage after parturition.

The results of pairwise intergroup differential analysis are shown in Figures 8C–E. As shown in Figure 8C (EL vs. PF), *Solibacillus* was the most significantly differential taxon, with its average relative abundance significantly higher in the PF stage than in the EL stage (*p* < 0.05), making it the most representative dominant genus in the pre-foaling period. In addition, *Lysinibacillus, Rikenellaceae_RC9_gut_group, Phascolarctobacterium, Prevotellaceae_UCG-004*, and *Quinella* were also significantly enriched in the PF stage (*p* < 0.05), with their abundances markedly decreased after parturition. In contrast, *Rummeliibacillus* was the core enriched genus in the EL stage, with its average relative abundance significantly higher in the EL stage than in the PF stage (*p* < 0.05), becoming one of the dominant genera in the early lactation period. Other taxa significantly upregulated in the EL stage included *Streptococcus, Candidatus_Saccharimonas, Chishuiella, Weissella, Devosia*, and *Dietzia* (*p* < 0.05).

As shown in Figure 8D (PoF vs. PF), *Rikenellaceae_RC9_gut_group, Lysinibacillus, Prevotella, Prevotellaceae_UCG-004, Phascolarctobacterium, Prevotellaceae_UCG-003*, Quinella, *hoa5-07d05_gut_group, Alloprevotella*, and *Brevibacillus* were significantly enriched in the PF stage (*p* < 0.05), and their abundances decreased markedly after parturition. In contrast, *Christensenellaceae_R-7_group, Akkermansia*, and *Candidatus_Saccharimonas* were the core enriched taxa in the PoF stage, with their relative abundances significantly higher in the PoF stage than in the PF stage (*p* < 0.05). Other taxa significantly upregulated in the PoF stage included *Phoenicibacter, Eubacterium, Chishuiella, Weissella, Marvinbryantia, Blautia, Coprococcus*, and *Anaerofustis* (*p* < 0.05).

As shown in Figure 8E (EL vs. PoF), *Chishuiella, UCG-004*, and *Dietzia* were significantly enriched in the EL stage (*p* < 0.05), with their relative abundances significantly higher than those in the PoF stage. In contrast, *Marvinbryantia, Coprococcus*, and *Eubacterium_hallii_group* were significantly enriched in the PoF stage (*p* < 0.05), and their relative abundances decreased significantly after entering the early lactation period.

### 3.9 LEfSe analysis of differential gut microbial taxa during the perinatal period

**Figure 9:**
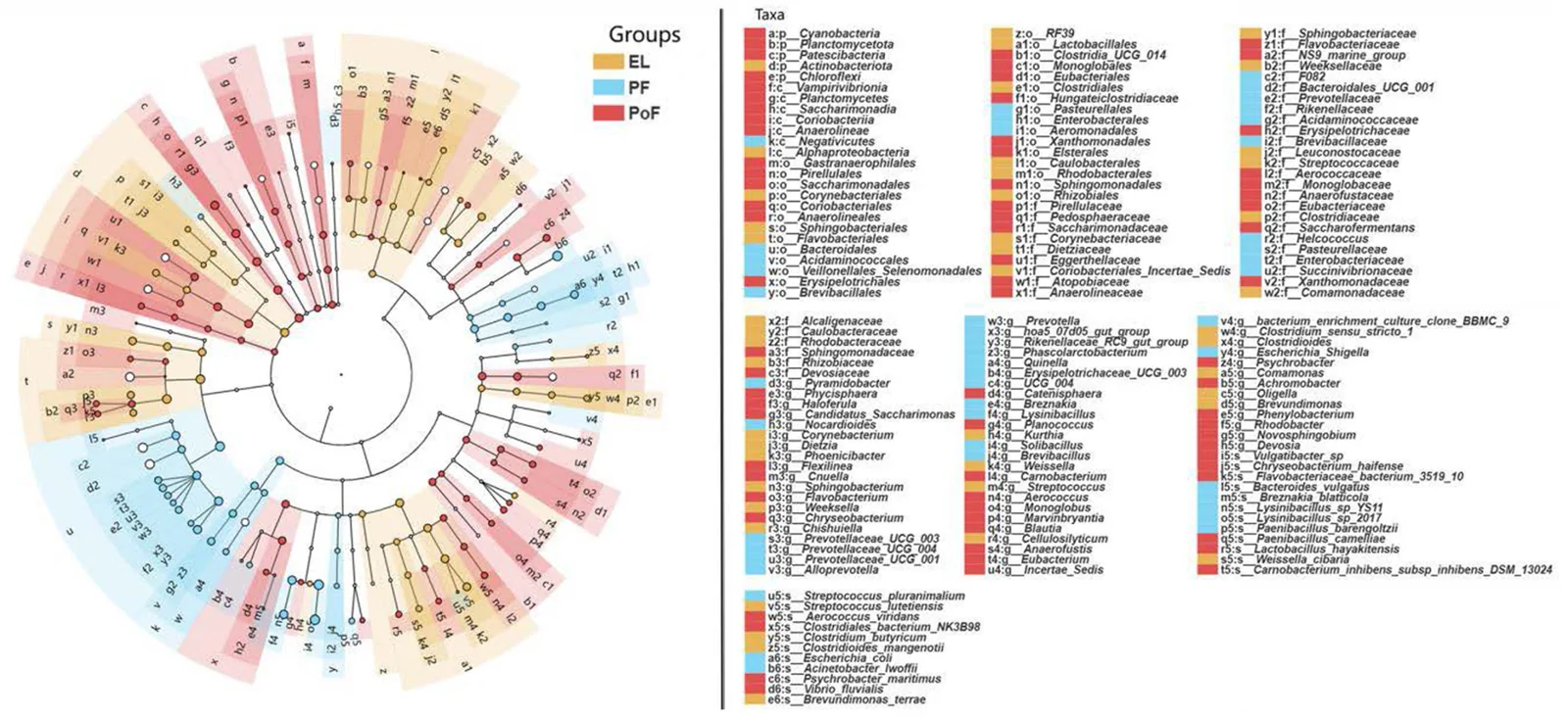
LEfSe cladogram of differential gut microbial taxa in perinatal Mongolian mares. Left panel: Evolutionary cladogram showing the hierarchical structure of gut microbiota, with nodes colored according to the stage in which the taxon is significantly enriched (blue: pre-foaling, PF; red: post-foaling, PoF; yellow: early lactation, EL). Right panel: Taxonomic annotation of all differentially enriched microbial taxa across all taxonomic ranks. *n* = 19.

Figure 9 is a cladogram generated by linear discriminant analysis effect size (LEfSe) analysis, which was used to systematically identify stage-associated discriminatory microbial features across three perinatal stages in Mongolian mares. The results showed that each stage was enriched with specific microbial taxa with distinct functional characteristics (Supplemental Table S5).

At the phylum level, LEfSe analysis identified five significantly stage-specific differentially enriched taxa. Specifically, the post-foaling (PoF) period was characterized by the specific enrichment of *Chloroflexi, Patescibacteria, Planctomycetota*, and *Cyanobacteria*, whereas the early lactation (EL) period was dominated by the core enriched phylum *Actinobacteriota*.

At the class level, LEfSe analysis identified seven significantly differentially enriched bacterial classes exhibiting distinct stage-specific distribution patterns throughout the perinatal period. The pre-foaling (PF) period was uniquely characterized by the specific enrichment of *Negativicutes* within the *Firmicutes* phylum. In contrast, the post-foaling (PoF) period harbored the largest number of differentially enriched classes, including *Anaerolineae, Planctomycetes, Saccharimonadia, Coriobacteriia*, and *Vampirivibrionia*. The early lactation (EL) period was specifically dominated by *Alphaproteobacteria* from the *Proteobacteria* phylum.

### 3.10 Functional prediction and differential analysis of gut microbiota during the perinatal period

**Figure 10:**
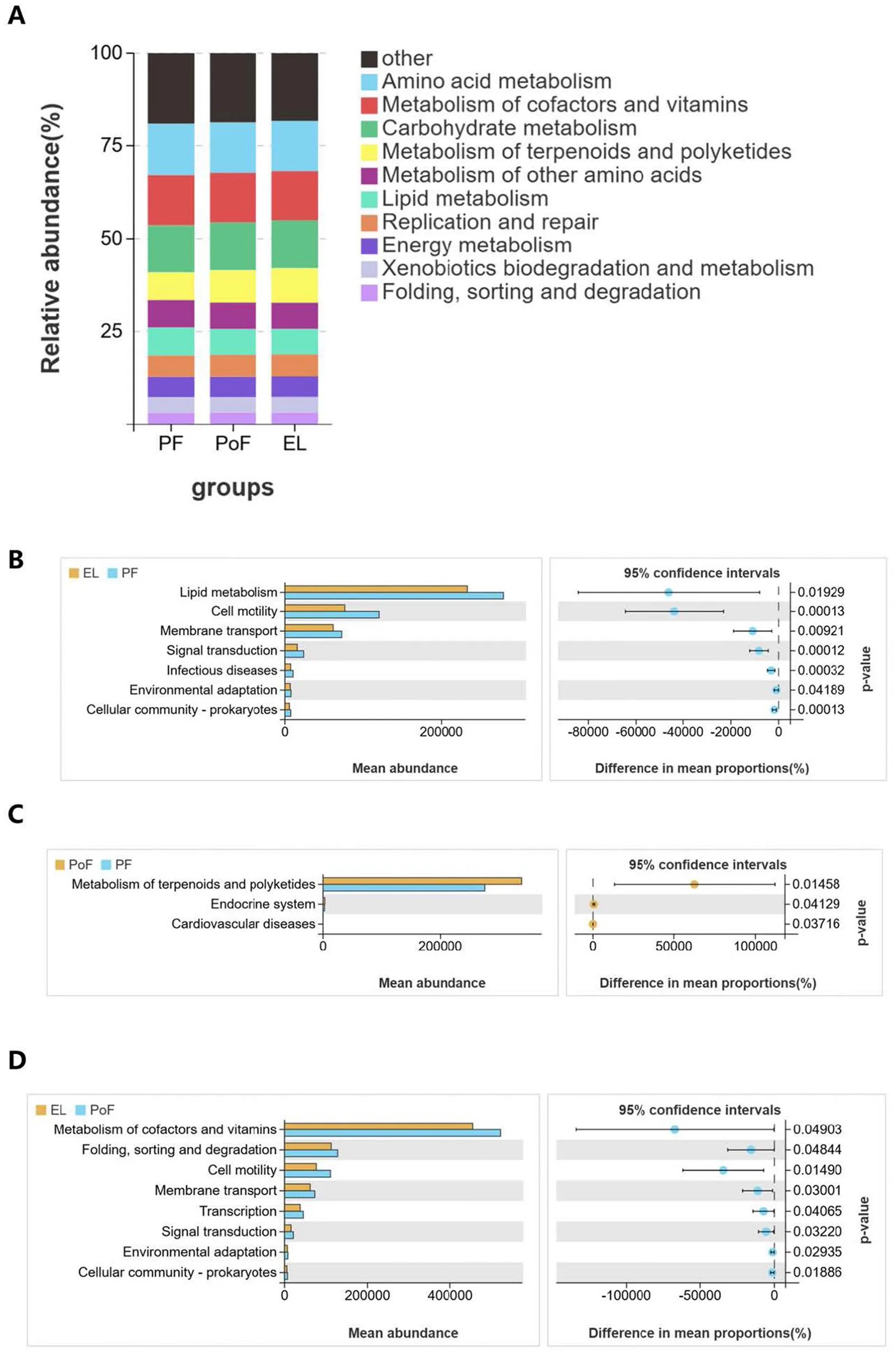
Functional prediction and differential pathway analysis of gut microbiota in perinatal Mongolian mares based on PICRUSt2 (*n* = 19). **(A)** Stacked bar chart showing the relative abundance of KEGG level 1 functional pathways in each stage; **(B)** Differential functional pathway analysis between the EL and PF stages using Welch's *t*-test; **(C)** Differential functional pathway analysis between the PoF and PF stages using Welch's *t*-test; **(D)** Differential functional pathway analysis between the EL and PoF stages using Welch's *t*-test.

Figure 10 presents the functional prediction results of gut microbiota during the perinatal period in Mongolian mares, including the overall composition of KEGG level 1 pathways and pairwise differential pathway analysis among the three stages.

As shown in Figure 10A, the overall functional profiles of gut microbiota were highly similar across the three stages. The core enriched metabolic pathways included carbohydrate metabolism, amino acid metabolism, metabolism of cofactors and vitamins, lipid metabolism, energy metabolism, and metabolism of terpenoids and polyketides.

The results of intergroup differential pathway analysis are shown in Figures 10B–D, with all statistical comparisons performed using Welch's *t*-test. Figure 10B demonstrated that compared with the PF stage, the lipid metabolism pathway was significantly enriched in the EL stage (*p* < 0.05), while the relative abundances of cell motility, membrane transport, signal transduction, infectious diseases, environmental adaptation, and cellular community—prokaryotes pathways were significantly decreased (*p* < 0.05). Figure 10C showed that compared with the PF stage, the metabolism of terpenoids and polyketides pathway was significantly increased in the PoF stage (*p* < 0.05). Figure 10D indicated that compared with the EL stage, the metabolism of cofactors and vitamins, folding, sorting and degradation, cell motility, membrane transport, transcription, signal transduction, environmental adaptation, and cellular community—prokaryotes pathway was significantly enriched in the PoF stage (*p* < 0.05).

### 3.11 Global characteristics of fecal metabolome during the perinatal period

**Figure 11:**
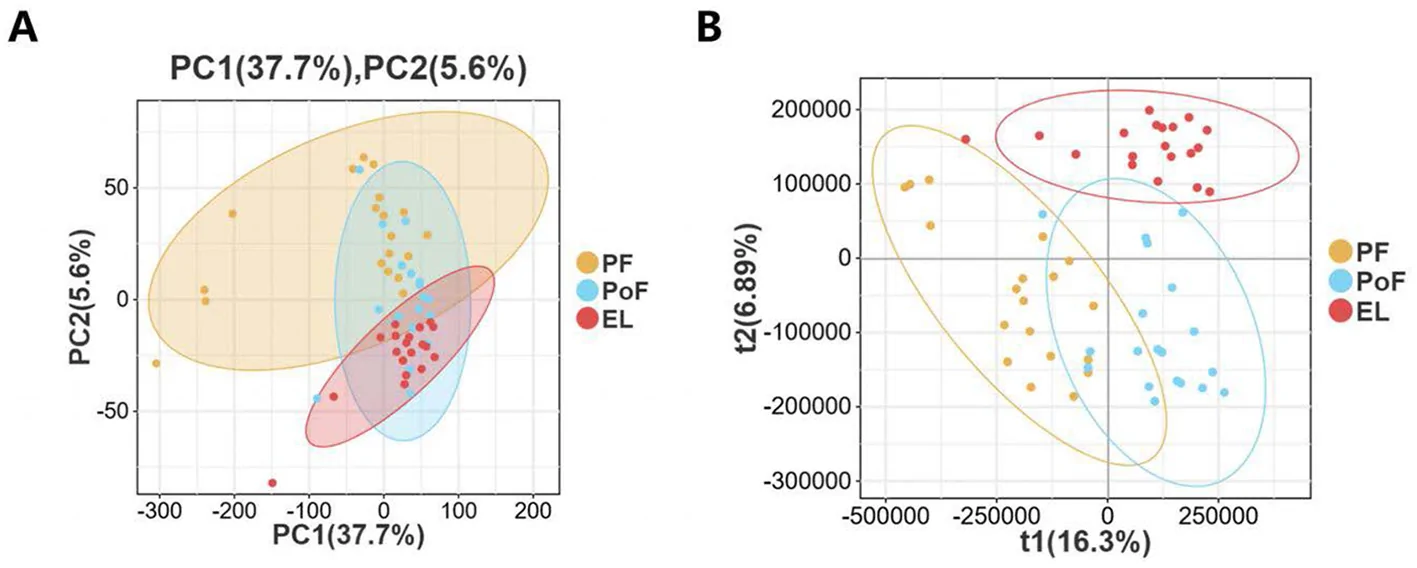
Global characteristics of fecal metabolome in perinatal Mongolian mares (*n* = 19). **(A)** Unsupervised principal component analysis (PCA) score plot showing the overall distribution of fecal metabolome profiles; **(B)** Supervised partial least squares discriminant analysis (PLS-DA) score plot showing the separation of metabolome profiles among stages.

Figure 11 presents the global differential characteristics of the fecal metabolome across three perinatal stages (PF, PoF, and EL) in Mongolian mares, visualized using unsupervised principal component analysis (PCA, Figure 11A) and supervised partial least squares discriminant analysis (PLS-DA, Figure 11B).

As shown in Figure 11A, the first principal component (PC1) explained 37.7% of the total metabolic variance, and the second principal component (PC2) explained 5.6% of the total variance in the PCA score plot. Samples from the three stages showed a clear gradient separation trend: PF samples were mainly concentrated on the left side of the PC1 axis, EL samples were mainly concentrated on the right side of the PC1 axis, while PoF samples were distributed between the two stages with partial overlap with both PF and EL stages, reflecting the gradual transition process of the metabolome from pregnancy to lactation during the perinatal period.

As shown in Figure 11B, the PLS-DA score plot further clearly demonstrated the metabolic differences among the three stages. The first principal component (t_1_) explained 16.3% of the variance, and the second principal component (t_2_) explained 6.89% of the variance. Compared with PCA, PLS-DA achieved better separation of the three stages of samples. PF, PoF, and EL stages each formed independent clusters with no significant overlap between stages, indicating that the fecal metabolomes of the three stages had significant global differences, and the lactation initiation process was accompanied by profound and specific metabolic remodeling.

### 3.12 Screening and distribution characteristics of differential metabolites during the perinatal period

**Figure 12:**
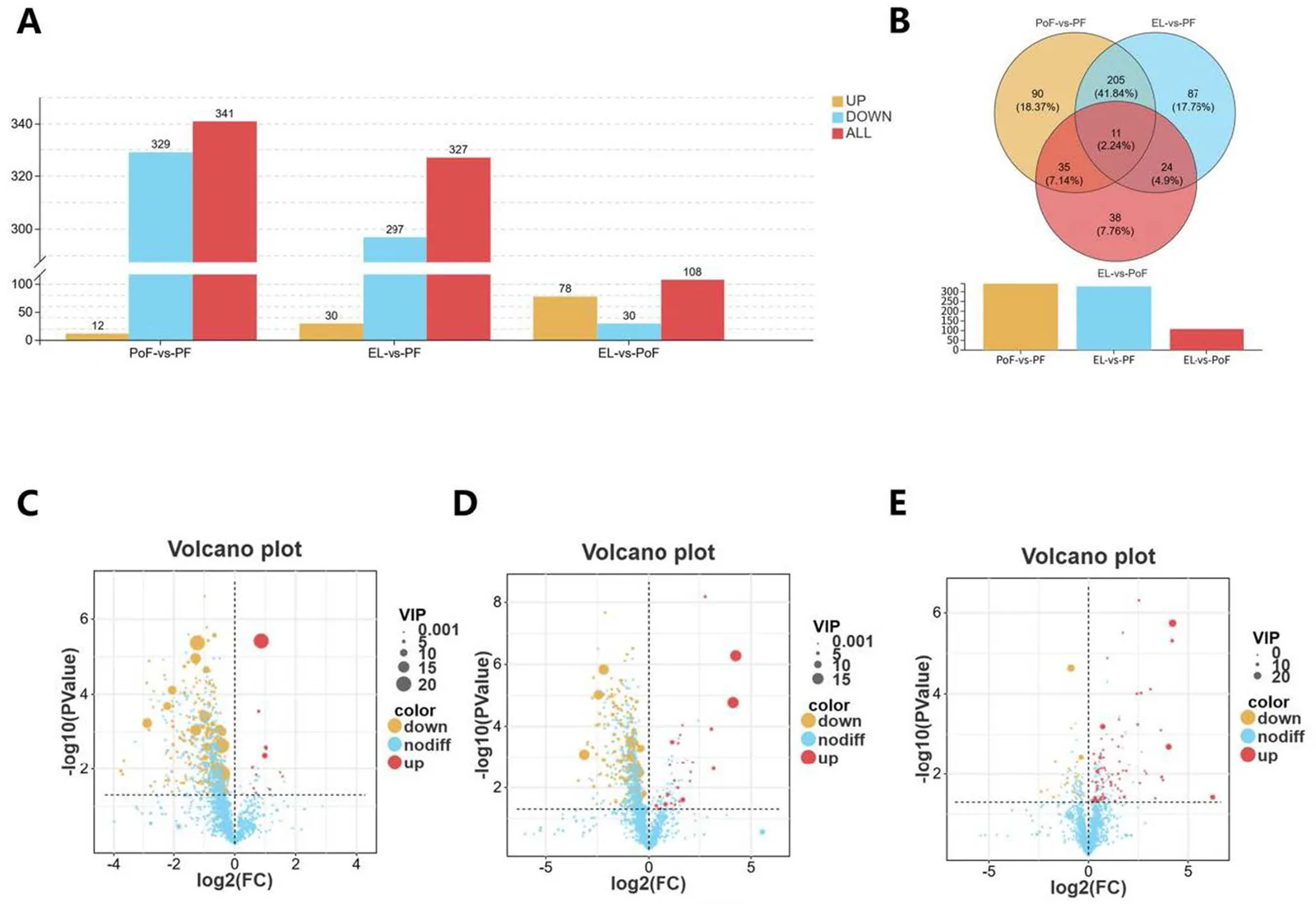
Screening and distribution characteristics of differential fecal metabolites in perinatal Mongolian mares. Differential metabolites were defined by VIP ≥ 1 from the OPLS-DA model and *p* < 0.05 from Student's *t*-test. **(A)** Bar chart showing the number of upregulated, downregulated and total differential metabolites in each pairwise comparison; **(B)** Venn diagram showing shared and unique differential metabolites among the three comparison stages, with the lower bar plot indicating the total number of differential metabolites in each comparison; **(C)** Volcano plot of differential metabolites between the PoF and PF stages; **(D)** Volcano plot of differential metabolites between the EL and PF stages; **(E)** Volcano plot of differential metabolites between the EL and PoF stages. n = 19.

Figure 12 systematically presents the quantity, distribution, and significance characteristics of differential fecal metabolites across three perinatal stages (PF, PoF, and EL) in Mongolian mares, including differential metabolite count statistics, Venn diagram analysis, and volcano plot visualization.

As shown in Figure 12A, a large number of differential metabolites were identified in pairwise comparisons among the three stages: a total of 341 differential metabolites were screened between the PoF and PF stages, of which 12 were significantly upregulated and 329 were significantly downregulated (Figure 12C); 327 differential metabolites were identified between the EL and PF stages, with 30 significantly upregulated and 297 significantly downregulated (Figure 12D); 108 differential metabolites were identified between the EL and PoF stages, including 78 significantly upregulated and 30 significantly downregulated (Figure 12E). The overall trend indicated that the vast majority of metabolites were downregulated after parturition (PoF and EL) compared with pre-foaling (PF), while metabolites in early lactation were mainly upregulated compared with post-foaling.

Venn diagram analysis in Figure 12B revealed that 11 core differential metabolites were shared among the three comparison stages, accounting for 2.24% of the total differential metabolites; 90 differential metabolites (18.37%) were unique to the PoF vs. PF stage, 87 (17.76%) were unique to the EL vs. PF stage, and 38 (7.76%) were unique to the EL vs. PoF stage. Notably, 205 differential metabolites (41.84%) were shared between the PoF vs. PF and EL vs. PF comparison stages, indicating that the two post-parturition stages shared a large number of common metabolic changes relative to pre-foaling. The bar plot below further showed that the total number of differential metabolites was highest in the PoF vs. PF stage, followed by the EL vs. PF stage, and lowest in the EL vs. PoF stage.

### 3.13 KEGG pathway enrichment analysis of differential metabolites

**Figure 13:**
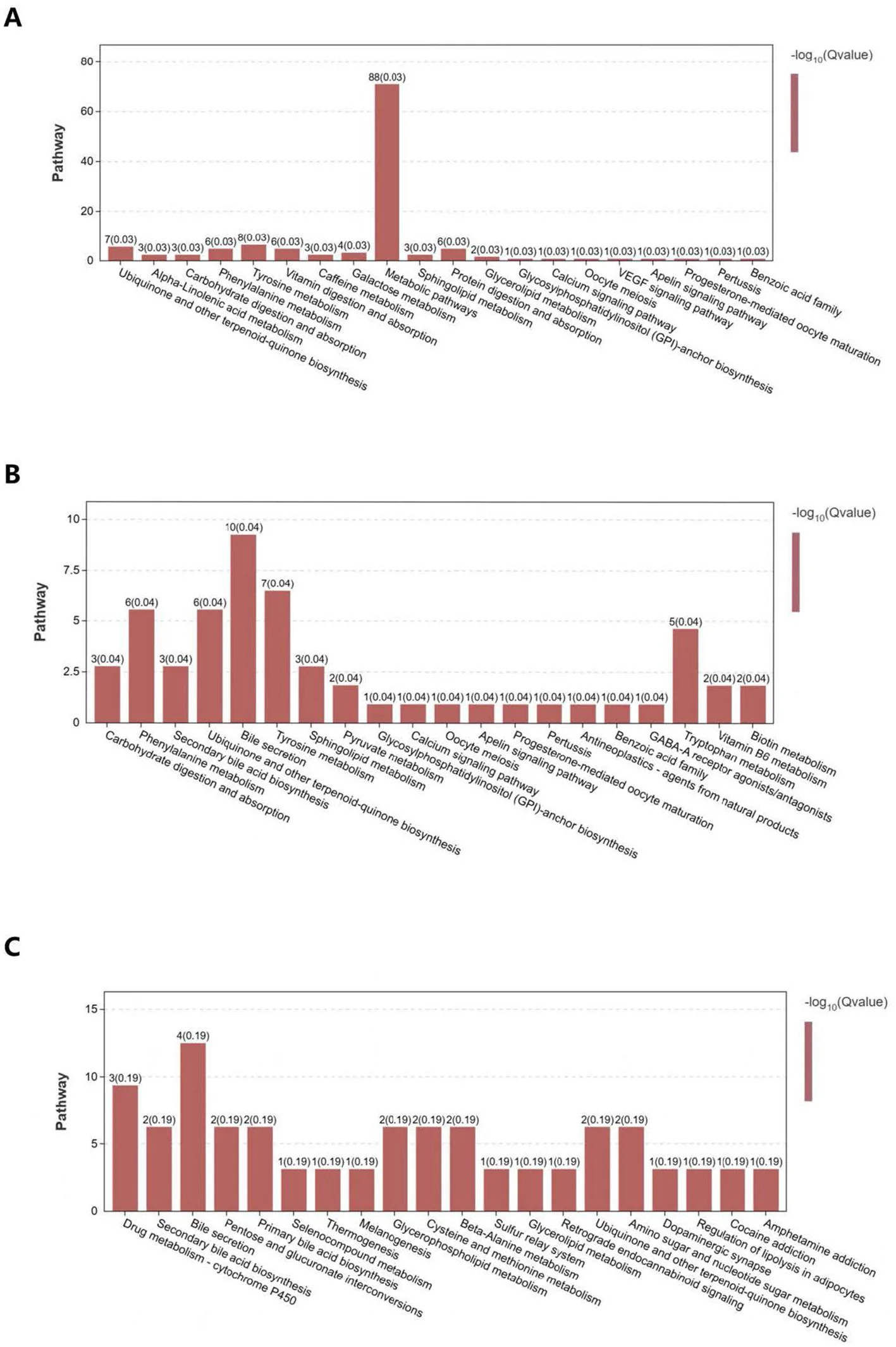
KEGG pathway enrichment analysis of differential fecal metabolites in perinatal Mongolian mares (*n* = 19). **(A)** Pathway enrichment analysis of differential metabolites between the PoF and PF stages; **(B)** Pathway enrichment analysis of differential metabolites between the EL and PF stages; **(C)** Pathway enrichment analysis of differential metabolites between the EL and PoF stages.

Figure 13 presents the KEGG pathway enrichment results of differential metabolites in pairwise comparisons among three perinatal stages (PF, PoF, and EL) in Mongolian mares. As shown in Figure 13A, a total of 17 significantly differential pathways were enriched between the PoF and PF stages (*p* < 0.05), among which the galactose metabolism pathway was one of the most significantly enriched pathways in the post-foaling period (*p* = 0.03). This fecal metabolic signature is temporally associated with parturition and cannot be directly interpreted as evidence of colostral lactose synthesis. Other enriched pathways may be related to perinatal energy metabolism and antioxidant adaptation.

As shown in Figure 13B, a total of 16 significantly differential pathways were enriched between the EL and PF stages (*p* < 0.05), among which the bile secretion pathway was one of the most significantly enriched pathways in the early lactation vs. pre-foaling comparison (*p* = 0.04). Other enriched pathways may be associated with perinatal lipid metabolism and immune adaptation.

As shown in Figure 13C, a total of 18 differential pathways were enriched between the EL and PoF stages, the bile secretion pathway showed a non-significant trend of enrichment in the EL vs. PoF comparison (*p* = 0.19), which did not reach statistical significance and should be interpreted cautiously. Other pathways may reflect gradual metabolic adaptation during early lactation.

### 3.14 Correlation analysis between gut microbiota and fecal differential metabolites

**Figure 14:**
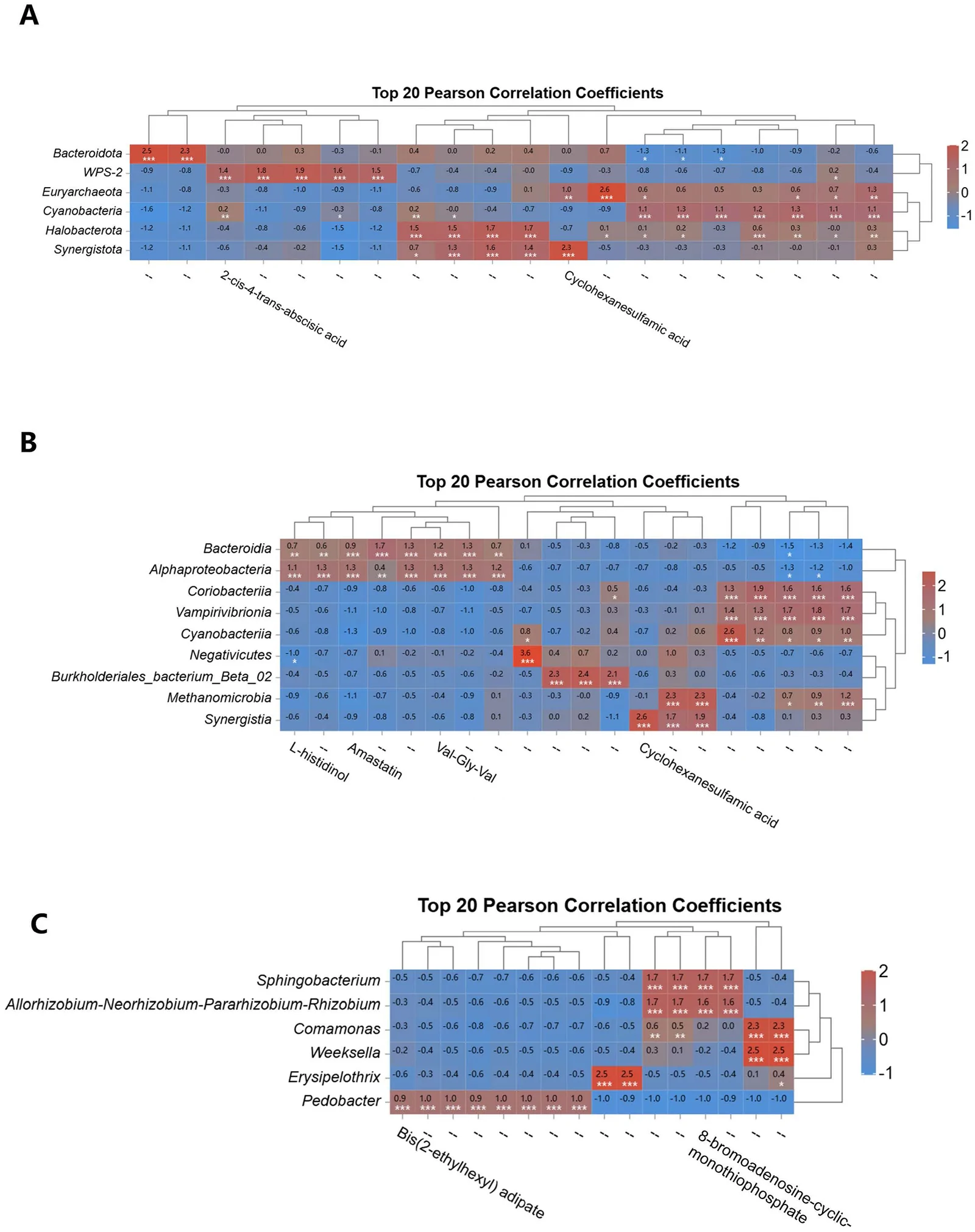
Correlation analysis between gut microbiota and differential fecal metabolites in perinatal Mongolian mares (*n* = 19). **(A)** Heatmap of the top 20 Pearson correlation coefficients between gut microbiota at the phylum level and differential metabolites; **(B)** Heatmap of the top 20 Pearson correlation coefficients between gut microbiota at the class level and differential metabolites; **(C)** Heatmap of the top 20 Pearson correlation coefficients between gut microbiota at the genus level and differential metabolites.

Figure 14 presents the top 20 Pearson correlation analysis results between gut microbiota and differential metabolites at the phylum, class, and genus levels during the perinatal period in Mongolian mares. As shown in Figure 14A (phylum level), *Euryarchaeota* and *Synergistota* showed positive correlations with cyclohexanesulfamic acid (*p* < 0.001). Notably, compounds including cyclohexanesulfamic acid, bis(2-ethylhexyl) adipate, 8-bromoadenosine cyclic monothiophosphate, and abscisic acid-related compounds may reflect dietary intake, plastic-derived contaminants, environmental exposure, analytical artifacts, or uncertain annotations, rather than endogenous equine or microbiota-derived metabolites. Their biological interpretation requires considerable caution. The *WPS-2* phylum showed a highly significant positive correlation with 2-cis-4-trans-abscisic acid (*p* < 0.001). The *Cyanobacteria* showed a significant positive correlation with 2-cis-4-trans-abscisic acid (*p* < 0.01).

As shown in Figure 14B (class level), *Bacteroidia* and *Alphaproteobacteria* showed highly consistent correlation characteristics: both were highly significantly positively correlated with L-histidinol, Amastatin, and Val-Gly-Val (*p* < 0.001). The Synergistia showed a highly significant positive correlation with cyclohexanesulfamic acid (*p* < 0.001).

As shown in Figure 14C (genus level), *Sphingobacterium* and *Allorhizobium*-*Neorhizobium*-*Pararhizobium*-*Rhizobium* all showed highly significant positive correlations with 8-bromoadenosine-cyclic-monothiophosphate (*p* < 0.001). In contrast, *Pedobacter* showed an extremely strong positive correlation with bis(2-ethylhexyl) adipate (*p* < 0.001).

## 4 Discussion

The perinatal period represents a critical physiological transition for female mammals, requiring coordinated adaptations in metabolism, endocrinology, and immunity to ensure successful parturition and lactation initiation (4, 23, 24). Although the regulatory role of the gut microbiota in lactation has been extensively studied in ruminants, its contribution to equine lactation remains poorly understood (9, 25, 26). This study performed parallel multi-omic profiling of serum physiology, fecal microbiota, and fecal metabolome across three key perinatal stages (pre-foaling, PF; post-foaling, PoF; and early lactation, EL) in Mongolian mares. Our results demonstrate that fecal microbiota and metabolome undergo significant stage-specific remodeling that is highly synchronized with host physiological adaptations. These findings are consistent with our hypothesis that fecal microbial remodeling is associated with perinatal physiological adaptation.

### 4.1 Adaptive changes in host physiological indices during the perinatal period

Consistent with the physiological demands of late pregnancy and lactation, serum biochemical and hormonal parameters exhibited clear stage-specific patterns. Pre-foaling mares had significantly higher concentrations of triglycerides, urea, calcium, and phosphorus, which may reflect metabolic preparation for late gestation and parturition (27–29). Following parturition, these parameters decreased continuously, which may be associated with the metabolic demands of early lactation (30, 31). Glucose concentrations peaked in the post-foaling period and remained elevated during early lactation, providing a critical energy source for parturition and lactogenesis (4, 32). Notably, aspartate aminotransferase (AST) levels increased significantly from pre-foaling to early lactation, while other liver function markers (alanine aminotransferase, alkaline phosphatase, and lactate dehydrogenase) remained stable. The observed AST elevation may be related to perinatal physiological stress, but the absence of parallel CK elevation limits definitive conclusions regarding muscle-specific damage (33, 34).

Reproductive hormone changes followed the expected physiological trajectory: progesterone concentrations dropped sharply after foaling, relieving the inhibitory effect on lactation, while estradiol peaked in the post-foaling period to promote mammary gland development (35, 36). Immune homeostasis remained largely stable throughout the perinatal period, with the exception of a significant increase in immunoglobulin A (IgA) during early lactation. This increase may be consistent with enhanced mucosal immune activation during the postpartum period. However, serum IgA cannot be directly equated with colostral IgA, and its contribution to foal passive immunity requires further validation (37, 38).

### 4.2 Stage-specific structural remodeling of the gut microbial community

The gut microbial community of Mongolian mares underwent significant structural and compositional changes during the perinatal period. Alpha diversity decreased significantly immediately after parturition and began to recover gradually during early lactation, but did not return to pre-foaling levels within the study period. This transient reduction in diversity has been reported in multiple mammalian species around parturition, including humans, cows, and pigs, and may be related to hormonal fluctuations, immune changes, and dietary shifts during the perinatal period (39–41). Beta diversity analysis revealed clear separation of microbial communities among the three stages, indicating that each perinatal period is characterized by a distinct gut microbiota profile.

At the phylum level, the post-foaling period was characterized by the enrichment of *Chloroflexi, Patescibacteria, Planctomycetota*, and *Cyanobacteria*, while the early lactation period was dominated by *Actinobacteriota*. These phyla are generally low-abundance taxa but may play important functional roles in specific physiological contexts. For example, *Actinobacteriota* are known to be involved in bile acid metabolism and immune regulation, and their enrichment during early lactation may support the increased lipid absorption and immune demands of lactation (42–44). At the class level, the pre-foaling period was uniquely enriched in *Negativicutes* and *Bacilli*, both of which are associated with carbohydrate fermentation and energy harvest (42, 45, 46). This enrichment is consistent with the increased energy storage requirements of late pregnancy (47–49). In contrast, the post-foaling period harbored the largest number of differentially enriched classes, including *Anaerolineae, Planctomycetes, Saccharimonadia, Coriobacteriia*, and *Vampirivibrionia*, while the early lactation period was dominated by *Alphaproteobacteria*.

Genus-level analysis identified distinct stage-associated taxa for each perinatal stage. Pre-foaling (PF) mares were enriched in *Solibacillus, Lysinibacillus*, and *Rikenellaceae_RC9_gut_group*, all of which are involved in carbohydrate metabolism (50–52). The post-foaling (PoF) period was characterized by the enrichment of *Akkermansia, Christensenellaceae_R-7_group*, and *Candidatus_Saccharimonas*. *Akkermansia* is a widely studied mucus-associated bacterium that has been linked to intestinal barrier maintenance in other mammalian species (53–55). Its increased abundance after parturition is consistent with a possible role in intestinal barrier homeostasis during perinatal adaptation, but this requires functional validation in horses (56, 57).

The early lactation stage was associated with increased relative abundances of *Rummeliibacillus, Streptococcus*, and *Candidatus_Saccharimonas*. Based on existing literature, these genera have been implicated in carbohydrate fermentation, fiber degradation, and immune modulation in the gut. However, genus-level 16S classification cannot establish species-specific activity or definitive functional roles (58–60). *Rummeliibacillus*, a spore-forming genus in the *Bacillaceae* family, was the most representative stage-associated microbial feature of the EL period. Previous studies have suggested that *Rummeliibacillus* may be involved in carbohydrate and fiber degradation in the gastrointestinal tract, which may be associated with the metabolic demands of lactation (61–64). For Mongolian mares grazing on low-quality forage under cold conditions, *Rummeliibacillus*-mediated energy extraction provides a critical adaptive advantage, directly supporting the high energy demands of lactation, which aligns with our observation of sustained high serum glucose concentrations in the EL period. Notably, *Rummeliibacillus* also exhibits stress resistance (65), enabling it to maintain metabolic activity even in the hindgut of mares exposed to extreme low temperatures, making it particularly well-adapted to the harsh rearing environment of Mongolian horses. *Streptococcus*, a core lactic acid bacterium in the equine gut, serves as the key immune regulatory taxon in the EL period. As a commensal genus consistently detected in the gut and mammary glands of healthy horses (42), *Streptococcus* regulates intestinal immune homeostasis by producing lactic acid to lower intestinal pH and inhibit pathogenic colonization, while also stimulating intestinal epithelial cells to secrete secretory IgA. This proposed function is consistent with our observation of higher serum IgA in early lactation. The entero-mammary migration of *Streptococcus* and its role in colostral immunity have been proposed in other species, but this mechanism has not been confirmed in horses (87). *Candidatus_Saccharimonas*, a representative genus of the Candidate Phyla Radiation (CPR) group *Patescibacteria*, is a specialized fiber-degrading taxon enriched in the EL period (66–68). The enrichment of *Candidatus_Saccharimonas* reflects a unique adaptive strategy of Mongolian mares: by recruiting specialized fiber-degrading bacteria, they maximize energy extraction from low-quality forage to support lactation without increasing feed intake. Collectively, the co-enrichment of these three genera suggests a potential synergistic functional network, though this proposed interaction remains to be formally validated. *Candidatus_Saccharimonas* is known to degrade refractory fiber to provide fermentation substrates in other systems (69, 70), *Rummeliibacillus* can convert such substrates into energy precursors (71), and *Streptococcus* contributes to immune homeostasis that supports lactation safety (72, 73). While this hypothetical cascade offers a plausible partial explanation for the strong lactation adaptation of Mongolian mares under harsh grazing conditions, direct experimental evidence for this exact functional axis in perinatal mares is still lacking.

To sum up, the stage-specific enrichment pattern of core genera observed in this study is broadly consistent with findings from perinatal studies in dairy cows and sows, where carbohydrate-fermenting taxa dominate late gestation and barrier-maintaining and lipid-metabolizing taxa increase after parturition. However, the magnitude of diversity decline and the specific dominant taxa differ between monogastric horses and ruminants, likely reflecting differences in digestive physiology and dietary structure. The entero-mammary axis hypothesis proposes that gut commensal bacteria or their metabolites can translocate to the mammary gland and regulate lactation physiology; while our data cannot confirm this translocation pathway in horses, the synchronous shifts in fecal microbiota and serum immune markers provide indirect support for this potential mechanism, which merits further targeted investigation.

### 4.3 Functional shifts in the fecal metabolome and enrichment of lactation-related pathways

Fecal metabolome analysis revealed profound and stage-specific metabolic remodeling during the perinatal period. Unsupervised principal component analysis (PCA) showed a clear gradient separation of samples from pre-foaling to early lactation, with post-foaling samples forming an intermediate cluster, reflecting the gradual transition from pregnancy to lactation. Supervised partial least squares discriminant analysis (PLS-DA) further confirmed the significant global differences in the metabolomes among the three stages. Notably, the vast majority of metabolites were downregulated after parturition compared with pre-foaling, while early lactation was characterized by an upregulation of metabolites relative to the post-foaling period. This pattern suggests that parturition induces a major metabolic shift, followed by a gradual adaptation to support lactation (4, 74).

KEGG pathway enrichment analysis identified distinct functional pathways associated with each stage transition. The galactose metabolism pathway was significantly enriched in the post-foaling vs. pre-foaling comparison. This fecal metabolic signature is temporally associated with the post-foaling period and may be related to perinatal carbohydrate metabolism changes, but cannot be directly interpreted as evidence of colostral lactose synthesis (75–77). Other enriched pathways in the post-foaling period included ubiquinone and other terpenoid-quinone biosynthesis, alpha-linolenic acid metabolism, and antioxidant pathways, which may reflect the increased oxidative stress associated with parturition (78, 79). In contrast, the bile secretion pathway was the most significantly enriched pathway in both the early lactation vs. pre-foaling and early lactation vs. post-foaling comparisons. Bile acids are involved in dietary lipid absorption, and enrichment of bile secretion-related metabolites may be associated with increased metabolic demands during early lactation (80, 81). Similar patterns have been observed in dairy cows, but the functional significance of these changes in horses requires further investigation (82, 83).

To sum up, compared with ruminant studies that report strong activation of milk fat synthesis-related pathways in early lactation, the enrichment of bile secretion-related features in this study is relatively mild and does not reach statistical significance in the EL vs. PoF comparison. This difference may be related to the lower lactation intensity and lower energy demand of horses compared with high-yielding dairy cows, and also reflects the limitation of inferring lactation function from fecal metabolic profiles.

### 4.4 Coordinated regulatory characteristics of gut microbiota and fecal metabolome during lactation adaptation

Consistent temporal remodeling was observed in both fecal microbiota and fecal metabolome across the three perinatal stages, which were coordinately associated with host serum metabolic, endocrine, and immune profiles during the perinatal transition. Stage-associated microbial taxa were correlated with distinct inferred metabolic profiles: carbohydrate-associated microbial taxa were more abundant in pre-foaling, which may support maternal energy storage, while post-partum enrichment of *Akkermansia* coincided with increased predicted galactose metabolism pathways, and this genus has been confirmed to participate in host energy metabolism regulation during post-partum physiological recovery in multiple mammalian species. In early lactation, microbial taxa potentially associated with lipid metabolism were enriched, along with enrichment of bile secretion-related metabolites. These coordinated changes may contribute to perinatal metabolic adaptation, and previous studies have verified its critical role in host lactation-related metabolic adaptation (64). Taxa belonging to *Actinobacteriota* are widely reported to modulate host protein and mineral metabolism during lactation (84, 85), while certain intestinal commensal bacteria can mediate the generation of bioactive small-molecule metabolites to regulate host systemic physiological homeostasis (86). The coordinated shifts in fecal microbiota and metabolome reflect stage-specific physiological adaptation during the perinatal transition in Mongolian mares.

Among the key correlation pairs, the positive correlation between mucus-associated Akkermansia and bile acid-related metabolites is consistent with a potential role in regulating intestinal barrier function and bile acid metabolism, which may indirectly support perinatal metabolic adaptation. The positive correlation between fiber-degrading genera such as Rummeliibacillus and carbohydrate metabolism-related metabolites aligns with their proposed role in enhancing energy extraction from forage. These consistent correlation patterns provide supporting evidence for the coordinated adaptation of gut microbiota and metabolome during the perinatal period, though causal relationships require further functional validation.

### 4.5 Limitations

This study has several limitations that should be noted. First, all samples were collected from a single breeding base and only one indigenous horse breed was included, which may limit the generalizability of the findings. Second, this is an observational associative study and cannot establish causal relationships between microbial/metabolic changes and perinatal physiological adaptation. Subsequent studies can expand the animal population and sampling sites, and combine functional verification experiments to further validate the universality and functional roles of the identified stage-associated features.

## 5 Conclusion

This study provides a systematic description of perinatal physiological, microbial, and metabolic changes in Mongolian mares. The identified fecal microbial taxa and metabolites are described as candidate stage-associated discriminatory features rather than validated biomarkers. The gut microbiota underwent profound structural remodeling across the transition period from late gestation to early lactation: pre-foaling mares were enriched with carbohydrate-degrading microbiota represented by *Solibacillus* and *Lysinibacillus* to facilitate maternal energy reserve accumulation; increased abundance of *Akkermansia* after parturition was associated with the perinatal transition, consistent with a potential role in intestinal barrier maintenance; early lactation was associated with increased abundances of *Rummeliibacillus, Streptococcus*, and *Candidatus_Saccharimonas*; these taxa may contribute to metabolic and immune adaptation, but their functional roles and synergistic effects require experimental validation. The coordinated shifts in fecal metabolome, including enrichment of galactose metabolism and bile secretion-related features, occurred in parallel with fecal microbial remodeling, in association with host perinatal physiological adaptation. The candidate stage-associated fecal microbial and metabolic signatures identified in this study provide valuable molecular references for germplasm conservation, perinatal nutritional regulation, and reproductive health management of indigenous horse breeds.

## Data Availability

The original contributions presented in the study are publicly available. This data can be found here: NCBI Sequence Read Archive (SRA), BioProject accession PRJNA1513058, BioSample accessions SAMN62404178, SAMN62404179, SAMN62404180.

## References

[1] Collier RJ Bauman DE Baumgard LH. Invited review: somatotropin and lactation biology. J Dairy Sci. (2025) 108:6538–49. doi: 10.3168/jds.2024-26177 40139351

[2] Agina OA. Haematology and clinical biochemistry findings associated with equine diseases - a review. Notulae Sci Biol. (2017) 9:1–21. doi: 10.15835/nsb919939

[3] Vervuert I. Feeding of the pregnant and lactating mare. In:Saastamoinen M, editor. Feeding and Management of Foals and Growing Horses. Cham: Springer (2023). doi: 10.1007/978-3-031-35271-3_1

[4] Bell AW. Regulation of organic nutrient metabolism during transition from late pregnancy to early lactation. J Anim Sci. (1995) 73:2804–19. doi: 10.2527/1995.7392804x 8582872

[5] Guo H Li J Wang Y Cao X Lv X Yang Z . Progress in research on key factors regulating lactation initiation in the mammary glands of dairy cows. Genes. (2023) 14:1163. doi: 10.3390/genes14061163 37372344 PMC10298202

[6] Zhang Q Chen D Ding H Li Q Yuan S Li H . Lactobacillus amylovorus extracellular vesicles mitigate mammary gland ferroptosis via the gut-mammary gland axis. NPJ Biofilms Microbiomes. (2025) 11:113. doi: 10.1038/s41522-025-00752-4 40544167 PMC12182568

[7] Kim CH. Complex regulatory effects of gut microbial short-chain fatty acids on immune tolerance and autoimmunity. Cell Mol Immunol. (2023) 20:341–50. doi: 10.1038/s41423-023-00987-1 36854801 PMC10066346

[8] Fischbach MA Sonnenburg JL. Eating for two: how metabolism establishes interspecies interactions in the gut. Cell Host Microbe. (2011) 10:336–47. doi: 10.1016/j.chom.2011.10.002 22018234 PMC3225337

[9] Weimer PJ. Redundancy, resilience, and host specificity of the ruminal microbiota: implications for engineering improved ruminal fermentations. Front Microbiol. (2015) 6:296. doi: 10.3389/fmicb.2015.00296 25914693 PMC4392294

[10] Morrison PK Newbold CJ Jones E Worgan HJ Grove-White DH Dugdale AH . The equine gastrointestinal microbiome: impacts of age and obesity. Front Microbiol. (2018) 9:3017. doi: 10.3389/fmicb.2018.03017 30581426 PMC6293011

[11] Zhao Y Li B Bai D Huang J Shiraigo W Yang L . Comparison of fecal microbiota of mongolian and thoroughbred horses by high-throughput sequencing of the V4 region of the 16S rRNA gene. Asian-Australas J Anim Sci. (2016) 29:1345–52. doi: 10.5713/ajas.15.0587 26954132 PMC5003997

[12] Marie-Etancelin C Déru V Carillier-Jacquin C Brulin L Estellé J Sanchez MP., et al. Review: the genetic architecture of host-microbiota interactions in livestock: a comprehensive review and critical appraisal. Animal. (2026) 20:101816. doi: 10.1016/j.animal.2026.101816 42033956

[13] Stewart HL Pitta D Indugu N Vecchiarelli B Engiles JB Southwood LL. Characterization of the fecal microbiota of healthy horses. Am J Vet Res. (2018) 79:811–9. doi: 10.2460/ajvr.79.8.811 30058849

[14] Liu Y Wang X He Q Wang G Wu Z Liu Q . Effects of dietary protein on weight gain, biochemical parameters, and gut microbiota in late-gestation grazing mongolian mares. Agriculture. (2026) 16:936. doi: 10.3390/agriculture16090936

[15] Aoki T Ishii M. Hematological and biochemical profiles in peripartum mares and neonatal foals (heavy draft horse). J Equine Veter Sci. (2012) 32:170–6. doi: 10.1016/j.jevs.2011.08.015

[16] Mariella J Pirrone A Gentilini F Castagnetti C. Hematologic and biochemical profiles in Standardbred mares during peripartum. Theriogenology. (2014) 81:526–34. doi: 10.1016/j.theriogenology.2013.11.001 24361129

[17] Banimehdi P Karimi-Dehkordi M Taktaz-Hafshejani T. Ultrasonographic appearance, reproductive hormone profile, and pregnancy rate in the Dareshoor mare. Comp Clin Pathol. (2025) 34:1083–9. doi: 10.1007/s00580-025-03715-5

[18] Guo H Kuang P Luo Q Cui H Deng H Liu H . Effects of sodium fluoride on blood cellular and humoral immunity in mice. Oncotarget. (2017) 8:85504–15. doi: 10.18632/oncotarget.20198 29156736 PMC5689626

[19] Dunn WB Broadhurst D Begley P Zelena E Francis-McIntyre S Anderson N . Procedures for large-scale metabolic profiling of serum and plasma using gas chromatography and liquid chromatography coupled to mass spectrometry. Nat Protoc. (2011) 6:1060–83. doi: 10.1038/nprot.2011.335 21720319

[20] Ritchie ME Phipson B Wu D Hu Y Law CW Shi W . Smyth, limma powers differential expression analyses for RNA-sequencing and microarray studies. Nucleic Acids Res. (2015) 43:e47. doi: 10.1093/nar/gkv007 25605792 PMC4402510

[21] Pang Z Chong J Zhou G de Lima Morais DA Chang L Barrette M . MetaboAnalyst 5.0: narrowing the gap between raw spectra and functional insights. Nucleic Acids Res. (2021) 49:W388–96. doi: 10.1093/nar/gkab382 34019663 PMC8265181

[22] Wang C Yang Y Chen J Dai X Xing C Zhang C . Berberine protects against high-energy and low-protein diet-induced hepatic steatosis: modulation of gut microbiota and bile acid metabolism in laying hens. Int J Mol Sci. (2023) 24:17304. doi: 10.3390/ijms242417304 38139133 PMC10744296

[23] Fowden AL Forhead AJ Ousey JC. Endocrine adaptations in the foal over the perinatal period. Equine Vet J Suppl. (2012) 130–9. doi: 10.1111/j.2042-3306.2011.00505.x 22594041

[24] Luo X Huang B Xu P Wang H Zhang B Lin L . The placenta regulates intrauterine fetal growth via exosomal PPARγ. Adv Sci. (2025) 12:e2404983. doi: 10.1002/advs.202404983 39951006 PMC12005745

[25] Monteiro HF Zhou Z Gomes MS Peixoto PMG Bonsaglia ECR Canisso IF . Rumen and lower gut microbiomes relationship with feed efficiency and production traits throughout the lactation of Holstein dairy cows. Sci Rep. (2022) 12:4904. doi: 10.1038/s41598-022-08761-5 35318351 PMC8940958

[26] Li M Zhu S Sun H Huo Y Cao Q Deng Z . Rumen microbiota modulates metabolic stress in high-yield dairy cows: insights from early to peak lactation. Microbiome. (2026) 14:61. doi: 10.1186/s40168-025-02318-0 41514445 PMC12879398

[27] Bazzano M Giannetto C Fazio F Arfuso F Giudice E Piccione G. Metabolic profile of broodmares during late pregnancy and early post-partum. Reprod Domest Anim. (2014) 49:947–53. doi: 10.1111/rda.12411 25251226

[28] Mazzo HC Wayne Nogueira CE Patten R Dos Reis Noronha H Da Silva GC Rosa Curcio B . Changes in cholesterol, triglycerides and body composition in pregnant mares. Acta Sci Vet. (2021) 49:110128. doi: 10.22456/1679-9216.110128

[29] Nasciutti NR Garcia FG da Silva ESM de Miranda RL Fontes LAR Rosa JB . Energy and mineral metabolism of peripartum mares and foals of the Quarter Horse breed. Vet Clin Pathol. (2021) 50:535–42. doi: 10.1111/vcp.13033 34873725

[30] Grummer RR Mashek DG Hayirli A. Dry matter intake and energy balance in the transition period. Vet Clin North Am Food Anim Pract. (2004) 20:447–70. doi: 10.1016/j.cvfa.2004.06.013 15471620

[31] Hui F Tong M Li S Zhao Y Guo X Guo Y . Effect of dietary energy level during late gestation on mineral contents in colostrum, milk, and plasma of lactating jennies. Animals. (2024) 14:2383. doi: 10.3390/ani14162383 39199917 PMC11350659

[32] Hoffman RM Kronfeld DS Cooper WL Harris PA. Glucose clearance in grazing mares is affected by diet, pregnancy, and lactation. J Anim Sci. (2003) 81:1764–71. doi: 10.2527/2003.8171764x 12854813

[33] Djuricic D Dobranic T Grizelj J Gracner D Harapin I Stanin D . Concentrations of total proteins and albumins, and AST, AP, CK and GGT activities in the blood serum Boer and Saanen goats during puerperium. Reprod Domest Anim. (2011) 46:674–7. doi: 10.1111/j.1439-0531.2010.01726.x 21114794

[34] Sookoian S Pirola CJ. Alanine and aspartate aminotransferase and glutamine-cycling pathway: their roles in pathogenesis of metabolic syndrome. World J Gastroenterol. (2012) 18:3775–81. doi: 10.3748/wjg.v18.i29.3775 22876026 PMC3413046

[35] Noden PA Oxender WD Hafs HD. Plasma luteinzing hormone, progestogens, and estrogens in mares during gestation, parturition, and first postpartum estrus (foal estrus). Am J Vet Res. (1978) 39:1964–7. doi: 10.2460/ajvr.1978.39.12.1964 749581

[36] da Silva AG Paulino MF da Silva Amorim L Rennó LN Detmann E de Moura FH . Performance, endocrine, metabolic, and reproductive responses of Nellore heifers submitted to different supplementation levels pre- and post-weaning. Trop Anim Health Prod. (2017) 49:707–15. doi: 10.1007/s11250-017-1248-1 28190129

[37] Reiter AS Reed SA. Lactation in horses. Anim Front. (2023) 13:96–100. doi: 10.1093/af/vfad003 37324210 PMC10266743

[38] Shokrollahi B Choi JY Won M Kim ET Lee SE Ham JS. Koumiss (fermented mare's milk) as a functional food: bioactive proteins, peptides, and future perspectives. Foods. (2025) 14:3954. doi: 10.3390/foods14223954 41300112 PMC12652487

[39] Huang X Gao J Zhao Y He M Ke S Wu J . Dramatic Remodeling of the Gut Microbiome Around Parturition and Its Relationship With Host Serum Metabolic Changes in Sows. Front Microbiol. (2019) 10:2123. doi: 10.3389/fmicb.2019.02123 31572329 PMC6751307

[40] Fu H He M Wu J Zhou Y Ke S Chen Z . Deep investigating the changes of gut microbiome and its correlation with the shifts of host serum metabolome around parturition in sows. Front Microbiol. (2021) 12:729039. doi: 10.3389/fmicb.2021.729039 34603257 PMC8484970

[41] Kim YH Kimura A Sugino T Sato S. Parturition and postpartum dietary change altered ruminal pH and the predicted functions of rumen bacterial communities but did not alter the bacterial composition in Holstein cows. Front Vet Sci. (2022) 9:948545. doi: 10.3389/fvets.2022.948545 36090180 PMC9458962

[42] Costa MC Arroyo LG Allen-Vercoe E Stämpfli HR Kim PT Sturgeon A . Comparison of the fecal microbiota of healthy horses and horses with colitis by high throughput sequencing of the V3-V5 region of the 16S rRNA gene. PLoS ONE. (2012) 7:e41484. doi: 10.1371/journal.pone.0041484 22859989 PMC3409227

[43] Wang J Shan J Zhang Y Shi Y Lu D Wu Y . Targeted metabolomics analysis for serum Helicobacter pylori-positive based on liquid chromatography-tandem mass spectrometry. Biomed Chromatogr BMC. (2023) 37:e5622. doi: 10.1002/bmc.5622 36898359

[44] Oke OE Fasasi LO Opowoye IO Akosile OA. The role of gut microbiota-derived metabolites in modulating poultry immunometabolism. Front Physiol. (2025) 16:1700406. doi: 10.3389/fphys.2025.1700406 41367394 PMC12682650

[45] Marchandin H Teyssier C Campos J Jean-Pierre H Roger F Gay B . Negativicoccus succinicivorans gen. nov, sp nov, isolated from human clinical samples, emended description of the family Veillonellaceae and description of *Negativicutes classis* nov, *Selenomonadales* ord nov and *Acidaminococcaceae* fam nov in the bacterial phylum Firmicutes. Int J Syst Evol Microbiol. (2010) 60:1271–9. doi: 10.1099/ijs.0.013102-0 19667386

[46] Argentino ICV Godoy MG Seldin L Jurelevicius D. Distribution of bacillota in water and sediments from aquatic environments. Microb Ecol. (2025) 88:3. doi: 10.1007/s00248-024-02482-0 39937305 PMC11821768

[47] Collado MC Isolauri E Laitinen K Salminen S. Distinct composition of gut microbiota during pregnancy in overweight and normal-weight women. Am J Clin Nutr. (2008) 88:894–9. doi: 10.1093/ajcn/88.4.894 18842773

[48] Heni AC Fackelmann G Eibner G Kreinert S Schmid J Schwensow NI . Wildlife gut microbiomes of sympatric generalist species respond differently to anthropogenic landscape disturbances. Anim Microbiome. (2023) 5:22. doi: 10.1186/s42523-023-00237-9 37024947 PMC10080760

[49] Zhang B Jiang X Yu Y Cui Y Wang W Luo H . Rumen microbiome-driven insight into bile acid metabolism and host metabolic regulation. ISME J. (2024) 18:wrae098. doi: 10.1093/ismejo/wrae098 38836500 PMC11193847

[50] Weese JS Holcombe SJ Embertson RM Kurtz KA Roessner HA Jalali M . Changes in the faecal microbiota of mares precede the development of post partum colic. Equine Vet J. (2015) 47:641–9. doi: 10.1111/evj.12361 25257320

[51] Lindroth KM Dicksved J Pelve E Båverud V Müller CE. Faecal bacterial composition in horses with and without free faecal liquid: a case control study. Sci Rep. (2021) 11:4745. doi: 10.1038/s41598-021-83897-4 33637818 PMC7910430

[52] Qin X Xi L Zhao L Han J Qu H Xu Y . Exploring the distinctive characteristics of gut microbiota across different horse breeds and ages using metataxonomics. Front Cell Infect Microbiol. (2025) 15:1590839. doi: 10.3389/fcimb.2025.1590839 40692682 PMC12277257

[53] Everard A Belzer C Geurts L Ouwerkerk JP Druart C Bindels LB . Cross-talk between Akkermansia muciniphila and intestinal epithelium controls diet-induced obesity. Proc Natl Acad Sci U S A. (2013) 110:9066–71. doi: 10.1073/pnas.1219451110 23671105 PMC3670398

[54] Xue C Li G Gu X Su Y Zheng Q Yuan X . Health and disease: akkermansia muciniphila, the shining star of the gut flora. Research. (2023) 6:0107. doi: 10.34133/research.0107 37040299 PMC10079265

[55] Li Z Luo Z Hu D. Assessing fecal microbial diversity and hormone levels as indicators of gastrointestinal health in reintroduced przewalski's horses (Equus ferus przewalskii). Animals. (2024) 14:2616. doi: 10.3390/ani14172616 39272401 PMC11393964

[56] Cairangzhuoma Yamamoto M Muranishi H Inagaki M Uchida K Yamashita K . Skimmed, sterilized, and concentrated bovine late colostrum promotes both prevention and recovery from intestinal tissue damage in mice. J Dairy Sci. (2013) 96:1347–55. doi: 10.3168/jds.2012-5701 23295115

[57] Gao Y Davis B Zhu W Zheng N Meng D Walker WA. Short-chain fatty acid butyrate, a breast milk metabolite, enhances immature intestinal barrier function genes in response to inflammation *in vitro* and *in vivo*. Am J Physiol Gastrointest Liver Physiol. (2021) 320:G521–30. doi: 10.1152/ajpgi.00279.2020 33085904 PMC8238162

[58] Feng G Fan X Liang Y Li C Xing J He Y . Genomic and transcriptional characteristics of strain *Rum-meliibacillus* sp. TYF-LIM-RU47 with an aptitude of directly producing acetoin from lignocellulose. Fermentation. (2022) 8:414. doi: 10.3390/fermentation8080414

[59] Açar Kuru Y Aksu S Gökler AF Somuncu EI Yassibaş E Ayyildiz F. Gut health for two: the critical role of probiotics and prebiotics in pregnancy and lactation. Curr Nutr Rep. (2025) 14:106. doi: 10.1007/s13668-025-00698-1 40952551

[60] Frolova SG Vatlin AA Pospelova I Mitkin NA Kulieva GA Pavshintsev VV. the role of danio rerio in understanding pollutant-induced gut microbiome dysbiosis in aquatic ecosystems. Toxics. (2025) 13:769. doi: 10.3390/toxics13090769 41012390 PMC12473707

[61] Poszytek K Karczewska-Golec J Dziurzynski M Stepkowska-Kowalska O Gorecki A Decewicz P . Genome-wide and functional view of proteolytic and lipolytic bacteria for efficient biogas production through enhanced sewage sludge hydrolysis. Molecules. (2019) 24:2624. doi: 10.3390/molecules24142624 31323902 PMC6680700

[62] Miguel M Lee SS Mamuad L Choi YJ Jeong CD Son A . enhancing butyrate production, ruminal fermentation and microbial population through supplementation with Clostridium saccharobutylicum. *J Microbiol Biotechnol*. (2019) 29:1083–95. doi: 10.4014/jmb.1905.05016 31216841

[63] Li Y Fang L Xue F Mao S Xiong B Ma Z . Effects of bamboo leaf extract on the production performance, rumen fermentation parameters, and rumen bacterial communities of heat-stressed dairy cows. Anim Biosci. (2021) 34:1784–93. doi: 10.5713/ab.20.0527 33561328 PMC8563258

[64] Ying Z Xie S Xiu Z Sun Y Yang Q Gao H . Under heat stress conditions, selenium nanoparticles promote lactation through modulation of rumen microbiota and metabolic processes in dairy goats. Sci Rep. (2025) 15:9063. doi: 10.1038/s41598-025-93710-1 40097638 PMC11914082

[65] Romão IR do Carmo Gomes J Silva D Vilchez JI. The seed microbiota from an application perspective: an underexplored frontier in plant-microbe interactions. Crop Health. (2025) 3:12. doi: 10.1007/s44297-025-00051-6 41649661 PMC12825913

[66] Brown CT Hug LA Thomas BC Sharon I Castelle CJ Singh A . Unusual biology across a group comprising more than 15% of domain Bacteria. Nature. (2015) 523:208–11. doi: 10.1038/nature14486 26083755

[67] Peñalver M Paradela A Palacios-Cuéllar C Pucciarelli MG García-Del Portillo F. Experimental evidence of d-glutamate racemase activity in the uncultivated bacterium Candidatus Saccharimonas aalborgensis. *Environ Microbiol*. (2024) 26:e16621. doi: 10.1111/1462-2920.16621 38558504

[68] Chiba N Limviphuvadh V Ng CH Koyagi R Kino Y Nakamura Y . Preliminary study of gut microbiome influence on black Ivory coffee fermentation in Asian elephants. Sci Rep. (2025) 15:40548. doi: 10.1038/s41598-025-24196-0 41253982 PMC12627549

[69] Guo J Mu R Li S Zhang N Fu Y Hu X. Characterization of the bacterial community of rumen in dairy cows with laminitis. Genes. (2021) 12:1996. doi: 10.3390/genes12121996 34946945 PMC8700892

[70] Liu Y Li X Diao Q Ma T Tu Y. *In silico* and *in vitro* studies revealed that rosmarinic acid inhibited methanogenesis via regulating composition and function of rumen microbiota. J Dairy Sci. (2024) 107:7904–17. doi: 10.3168/jds.2024-24970 38851580

[71] Tan HY Chen SW Hu SY. Improvements in the growth performance, immunity, disease resistance, and gut microbiota by the probiotic *Rummeliibacillus stabekisii* in Nile tilapia (*Oreochromis niloticus*). Fish Shellfish Immunol. (2019) 92:265–75. doi: 10.1016/j.fsi.2019.06.027 31202962

[72] Lin PH Chen SW Wen ZH Hu SY. Administration of the potential probiotic *Paenibacillus ehimensis* NPUST1 enhances expression of indicator genes associated with nutrient metabolism, growth and innate immunity against *Aeromonas hydrophila* and *Streptococcus indie* infections in zebrafish (*Danio rerio*). Fishes. (2022) 7:386. doi: 10.3390/fishes7060386

[73] François AC Cesarini C Taminiau B Renaud B Kruse CJ Boemer F . Unravelling faecal microbiota variations in equine atypical myopathy: correlation with blood markers and contribution of microbiome. Animals. (2025) 15:354. doi: 10.3390/ani15030354 39943124 PMC11815872

[74] Favorit V Hood WR Kavazis AN Skibiel AL. Graduate student literature review: mitochondrial adaptations across lactation and their molecular regulation in dairy cattle. J Dairy Sci. (2021) 104:10415–25. doi: 10.3168/jds.2021-20138 34218917 PMC13317002

[75] Bionaz M Periasamy K Rodriguez-Zas SL Everts RE Lewin HA Hurley WL . Old and new stories: revelations from functional analysis of the bovine mammary transcriptome during the lactation cycle. PLoS ONE. (2012) 7:e33268. doi: 10.1371/journal.pone.0033268 22428004 PMC3299771

[76] Shi L Zhang L Wang L Liu X Gao H Hou X . Identifying long non-coding RNAs and characterizing their functional roles in swine mammary gland from colostrogenesis to lactogenesis. Anim Biosci. (2022) 35:814–25. doi: 10.5713/ab.21.0308 34727649 PMC9066039

[77] Xia W Liu Y Loor JJ Bionaz M Jiang M. Dynamic profile of the yak mammary transcriptome during the lactation cycle. Animals. (2023) 13:1710. doi: 10.3390/ani13101710 37238139 PMC10215676

[78] Martuzzi F Bresciani C Simoni M Basini G Quarantelli A Righi F. Evaluation of the oxidative status of periparturient mares supplemented with high amount of α-tocopherol. Ital J Anim Sci. (2019) 18:1404–9. doi: 10.1080/1828051X.2019.1677518

[79] Li Q Gu L Song J Li C Zhang Y Wang Y . Physiological and transcriptome analyses highlight multiple pathways involved in drought stress in Medicago falcata. *PLoS ONE*. (2022) 17:e0266542. doi: 10.1371/journal.pone.0266542 35390072 PMC8989214

[80] Li Q Liang R Li Y Gao Y Li Q Sun D . Identification of candidate genes for milk production traits by RNA sequencing on bovine liver at different lactation stages. BMC Genet. (2020) 21:72. doi: 10.1186/s12863-020-00882-y 32646377 PMC7346489

[81] Li Z Guo W Chu Y Tan X Wang H Feng J . Integrated transcriptome and metabolome analyses unraveled critical roles of small intestine during the weaning period of Vespertilio sinensis. BMC Genomics. (2025) 26:587. doi: 10.1186/s12864-025-11784-7 40596826 PMC12211977

[82] Zhang J Zhang X Liu H Wang P Li L Bionaz M . Altered bile acid and correlations with gut microbiome in transition dairy cows with different glucose and lipid metabolism status. J Dairy Sci. (2024) 107:9915–33. doi: 10.3168/jds.2024-24658 38908707

[83] Li L Li J Liu Z Jin Z Wang M Wu Y . Effects of supplementing bile acids on the production performance, fatty acid and bile acid composition, and gut microbiota in transition dairy cows. J Anim Sci Biotechnol. (2025) 16:83. doi: 10.1186/s40104-025-01207-8 40506720 PMC12160099

[84] Tsifintaris M Sitmalidis M Tokamani M Anastasiadi C Georganta M Tsochantaridis I . Analysis of human milk microbiota in northern greece by comparative 16S rRNA sequencing vs. local dairy. Anim Nutr. (2024) 16:2175. doi: 10.3390/nu16142175 39064618 PMC11280067

[85] Ding Z Xu Y Wang Y Liu M Zhu P Cui K . Host-driven remodeling of rumen microbiota supports lactation metabolism in buffalo. Front Microbiol. (2025) 16:1617388. doi: 10.3389/fmicb.2025.1617388 40636496 PMC12237897

[86] Hou S Yu J Li Y Zhao D Zhang Z. Advances in fecal microbiota transplantation for gut dysbiosis-related diseases. Adv Sci. (2025) 12:e2413197. doi: 10.1002/advs.202413197 40013938 PMC11967859

[87] Galan JE Timoney JF Lengemann FW. Passive transfer of mucosal antibody to *Streptococcus equi* in the foal. Infect Immun. (1986) 54:202–6. doi: 10.1128/iai.54.1.20-206.1986 3531013 PMC260137

